# Exposure to Environmental and Occupational Particulate Air Pollution as a Potential Contributor to Neurodegeneration and Diabetes: A Systematic Review of Epidemiological Research

**DOI:** 10.3390/ijerph15081704

**Published:** 2018-08-09

**Authors:** Eirini Dimakakou, Helinor J. Johnston, George Streftaris, John W. Cherrie

**Affiliations:** 1 Institute of Biological Chemistry, Biophysics and Bioengineering, Heriot-Watt University, Edinburgh EH14 4AS, UK; ed25@hw.ac.uk (E.D.); H.Johnston@hw.ac.uk (H.J.J.); 2 Maxwell Institute for Mathematical Sciences, School of Mathematical and Computer Sciences, Heriot-Watt University, Edinburgh EH14 4AS, UK; G.Streftaris@hw.ac.uk; 3 Institute of Occupational Medicine (IOM), Riccarton, Edinburgh EH14 4AP, UK

**Keywords:** type 2 diabetes, neurodegeneration, cognitive function, dementia, Alzheimer’s disease, Parkinson’s disease, air pollution, particulate matter, occupational, epidemiology

## Abstract

It has been hypothesised that environmental air pollution, especially airborne particles, is a risk factor for type 2 diabetes mellitus (T2DM) and neurodegenerative conditions. However, epidemiological evidence is inconsistent and has not been previously evaluated as part of a systematic review. Our objectives were to carry out a systematic review of the epidemiological evidence on the association between long-term exposure to ambient air pollution and T2DM and neurodegenerative diseases in adults and to identify if workplace exposures to particles are associated with an increased risk of T2DM and neurodegenerative diseases. Assessment of the quality of the evidence was carried out using the GRADE system, which considers the quality of the studies, consistency, directness, effect size, and publication bias. Available evidence indicates a consistent positive association between ambient air pollution and both T2DM and neurodegeneration risk, such as dementia and a general decline in cognition. However, corresponding evidence for workplace exposures are lacking. Further research is required to identify the link and mechanisms associated with particulate exposure and disease pathogenesis and to investigate the risks in occupational populations. Additional steps are needed to reduce air pollution levels and possibly also in the workplace environment to decrease the incidence of T2DM and cognitive decline.

## 1. Introduction

Globally, there is an estimated 2.3 million deaths every year attributed to work, with 86.9% of these attributed to work-related diseases [1]. In Britain, deaths from workplace exposure to chemicals, dusts, and fibres have increased over the last 40 years, with currently around 13,000 premature deaths each year from occupational lung disease and cancer [2]. The non-occupational environment can also have detrimental impacts on health. More specifically, individuals are exposed to many risk factors in their homes and in the environment such as air pollution, chemicals, ionising and non-ionising radiation, and noise. Exposure to air pollution is a particular concern to the public; with 91% of the world’s population living in areas where air quality is poor and exceeds World Health Organisation (WHO) guideline limits [3].

Overall occupational and environmental exposures account for around 12.5% of the total global mortality each year. Within this, it is estimated that air pollution is responsible for around 3.2% of the disease burden, which, according to WHO, accounts for an estimated 4.2 million deaths per year worldwide due to stroke, heart disease, lung cancer, and chronic respiratory diseases [3,4]. Outdoor air pollution contributes about 40,000 early deaths each year in the UK [5].

Ambient particulate air pollution, such as PM_10_, has been a public health concern in Britain since the ‘London Smog’ in 1952, when increased mortality and morbidity were reported soon after the event [6]. Similar incidents have occurred in Belgium (1930) and in Pennsylvania (1948) [7]. PM_10_ is particulate matter 10 micrometres or less in diameter and has a complex composition, with the ultrafine particle component held principally accountable for eliciting much of the toxicity associated with PM_10_ exposure [8,9]. Epidemiological studies indicate links between PM_10_ and PM_2.5_ with both short-term and long-term health effects [10]. Furthermore, both short-term (e.g., hours) and long-term exposure (e.g., months, years) can increase morbidity and mortality [11]. The link between PM_10_ and adverse health effects was first established in the 1990s, where epidemiological studies found a positive association between the level of particulate air pollution in cities and increased morbidity and mortality rates in both adults and children, with adverse health effects being manifested predominantly in susceptible individuals who had pre-existing pulmonary or cardiovascular disease [12,13]. It is now widely accepted that exposure to PM_10_ may cause or exacerbate allergic respiratory diseases (e.g., asthma) [14], chronic obstructive pulmonary disease, pneumonia, and cardiovascular disease [15] and is also defined as a definite carcinogen by International Agency for Research on Cancer (IARC) [16]. The cellular and molecular mechanisms underlying the toxicity of inhaled particles has been extensively investigated, and hypothesised to be driven by the stimulation of inflammation and/or oxidative stress [9].

More recently, there has also been evidence from epidemiological and laboratory studies that long-term exposure to particulate air pollution is associated with T2DM [17,18,19,20,21,22,23,24,25], dementia [26,27,28], and other neurodegenerative diseases, such as Alzheimer’s disease (AD) and Parkinson’s disease (PD) [28,29]. The ability of inhaled particles to stimulate adverse effects in extrapulmonary sites has been reviewed previously [30], and likely to manifest due to (i) the translocation of particles from the lung to other target sites, or (ii) release of mediators (e.g., cytokines) from the lung which act systemically [30,31].

The WHO estimates that 422 million people worldwide have diabetes, 47 million people are living with dementia and more than 10 million people are living with PD with increasing incidence expected over coming years.

As the world’s population is ageing, dementia has become a major public health concern. Most cases of neurological diseases are sporadic, and thus identification that particulate air pollution may be an important environmental risk factor may help better understand the aetiology of neurodegenerative disorders.

Diabetes is a metabolic disease, with different types (type 1 and type 2). Type 1 is often diagnosed in childhood and cannot be controlled without taking insulin, whereas type 2 is usually diagnosed in over 30 year olds and can generally be controlled with medication, or diet and exercise. Increasing evidence indicates that environmental exposures may cause diabetes and more recently T2DM [32].

In addition to particle exposure via the environment, workplace exposures to particles can also occur. Workplace exposures can include a range of fine dusts and diesel exhaust. Accordingly, it is possible that exposure to particles in an occupational setting may also increase risk of T2DM and neurodegenerative disease.

The aim of this review is to systematically explore the association between long-term exposure (years/decades) to airborne particulate matter, both from ambient environmental pollutants and workplace exposures, and T2DM and cognitive impairment; earlier onset of greater than age-related decline and/or neurodegeneration in adults. The information from the review is discussed in relation to occupational exposure to particulate matter and fumes, and risk of dementia and diabetes. We hypothesise that exposure to ambient fine airborne particles is associated with an increased risk of neurodegenerative conditions and T2DM, and that these risks also apply to workplace exposures to particulate matter.

## 2. Methods

### 2.1. Search Strategy and Study Selection

PubMed, Web of Science, Scopus, and Google Scholar were searched (last search, March 2018) for studies using different combinations of search terms, related to diabetes (‘diabetes mellitus’, ‘type 2 diabetes mellitus’, ‘diabetes mellitus’, and ‘insulin resistance’), neurodegenerative diseases (‘dementia’, ‘neurodegeneration’, ‘Alzheimer’s Disease’, ‘Parkinson’s Disease’, ‘cognitive decline’, ‘cognitive impairment’, and ‘neurocognitive disorders’), air pollution (‘air pollution’, ‘outdoor air pollution’, ‘traffic-related air pollution’, ‘air pollutants’, ‘ambient air pollution’, and ‘environmental pollution’), airborne particles (‘particulate matter’, ‘PM_10_’, ‘PM_2.5’_, ‘fine particulate matter’, ‘airborne particulate matter’, ‘ambient particulate matter, ‘ultrafine particles’, ‘black carbon’, ‘black smoke’, and ‘elemental carbon’), and finally occupational exposure (‘occupational exposure’, ‘work exposure’, ‘employment exposure’, and ‘exposure at workplace’).

**Figure 1:**
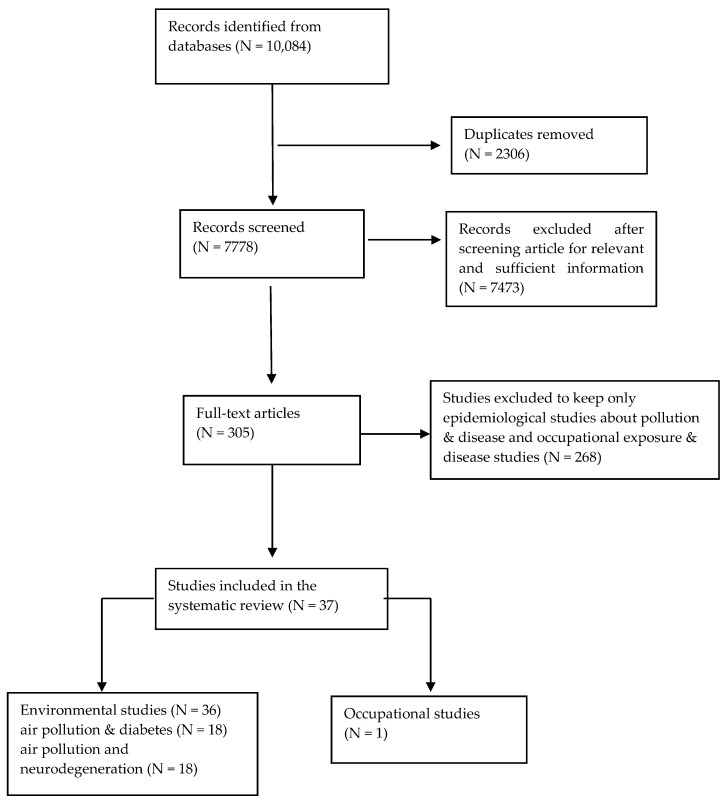
Flow chart of the literature search.

There were five combinations of search. First, air pollution and airborne particle terms were combined with diabetes terms and then with neurodegeneration disease terms. The third and fourth searches combined occupational exposure and diabetes and then neurodegeneration diseases, while the fifth search was a combination of diabetes and neurodegeneration disease terms. These five categories identified 10,084 articles. After de-duplication 7778 articles remained. First titles and then abstracts were screened for eligibility and potentially relevant articles were retrieved as full texts. The target population was adults, therefore all the studies that examined the associations in children were excluded. Articles were also excluded if they focused on pulmonary disease, chronic obstructive pulmonary disease (COPD), cardiovascular disease, lung disease, atherosclerosis, type-1 diabetes, vascular dementia, chemical exposures, organic solvents and pollutants, metals, smoking, asthma, birth outcomes, children, hypertension, different types of cancer, organic dust, bio-aerosols, allergic respiratory diseases, myocardial infraction, multiple sclerosis, diet, stroke, disease because of virus, asbestos, short-term (months) exposure to particles, pesticides, magnetic fields, welding fumes, bacterial inflammation, ozone, and nitric oxides. For articles without an abstract or enough information in the abstract to make a decision, the full text, and where necessary supplementary materials, were reviewed before a decision was made. We ended with 305 articles, separated into the five categories, according to the combinations described above. However, for practical reasons and to be able to compare the articles within one systematic review, we took into account only the epidemiological studies (toxicology studies and reviews were excluded), and we constructed two categories of publications for pollution and disease (‘air pollution/airborne particles and diabetes’ and ‘air pollution/airborne particles and neurodegenerative diseases’). In addition, two categories for occupational exposure and disease were included in the final appraisal. We ended up with 36 environmental epidemiological studies and only one occupational study (Figure 1).

### 2.2. Evaluation of the Systematic Review and Quality of the Studies

The internationally recognised GRADE system for the scientific evaluation of the quality of the identified studies was used to appraise the quality of the studies identified [33]. This system was developed for assessing interventions in health care contexts, and it has also been adapted to epidemiological studies [34,35]. In our case, an environmental/occupational factor or exposure can replace the clinical intervention [35]. The GRADE system classifies the quality of evidence as high, moderate, low, and very low and according to GRADE, evidence based on randomised controlled trials begins as ‘high quality’, and observational studies start as ‘low quality’. All of the cohort and case-control studies epidemiological studies identified for this study were observational. A well-designed observational study can be upgraded and provide a quality grading similar to a randomized controlled trial (RCT), and an RCT can be downgraded if certain aspects are missing. GRADE looks at studies individually, but also considers the whole evidence base.

The following aspects of quality were considered in this study [36]:

1. Representativeness and size of the study sample, as well as the duration of follow-up period. High number of participants, many years of follow-up, no attrition and lost during follow-up and participants that were members of a bigger cohort, could upgrade the quality of the studies.
2. Publication bias or other kinds of bias, such as selection bias, misclassifications, selective reporting, or conflict of interest diminish the quality of a study. The quality may be downgraded if authors presented incomplete or selectively reported the tested hypothesis, compared to their aim and objectives. If there was no declaration of funder and involvement of the funding body in the research or if the authors had indicated a conflict of interest the quality was also downgraded.
3. Potential confounders such as age, sex, physical activity, education level, alcohol intake, smoking status, socioeconomic status (SES) should have been considered. There is a need of an adequate control of confounding and adjustment in the statistical analysis. If more appropriate potential confounders were taken into account, then the quality of the study could be upgraded.
4. For both exposure and outcome assessment, standardized and validated methods were required. Well established methods enable the comparison between studies and as a result they can contribute to higher quality rating.

This systematic review follows the PRISMA guidelines (Appendix A).

## 3. Results

The available research uses many different methodological designs to explore the associations between environmental or occupational particle exposures and the health-related outcomes. The approaches mostly quantify particle exposures using various measures of environmental pollution, and the outcome measures are quite disparate.

### 3.1. Air Pollution and Neurodegeneration

The characteristics of all the included studies focused on investigating the relationship between air pollution exposure and dementia are summarized in Table 1 and Table 2. Of these, 10 studies were cohort studies, 4 studies were cross-sectional, 1 case-control, 2 nested case-control studies, and 1 was a time-series analysis. These were all recent studies, conducted after 2008, in North America (US, *n* = 10, Canada, *n* = 1), Europe (Germany, *n* = 3 and UK, *n* = 1), and Asia (Taiwan, *n* = 3). For the cohort studies the range of the follow up periods ranged from 5 to 30 years.

Most studies (17 out of 18) examined airborne particulate matter, four studies also included NO_x_ and two studies considered both PM and traffic noise or proximity to a major road. Five studies quantified black carbon (BC) as a measure of PM. Exposure estimates—assessments based on measurement data—were mostly based on data from air monitors at fixed monitoring sites, using either the participant’s postcode [26,37], address of residence [38,39,40,41,42], county [43] or the community [44]. Half of the studies (9 out of 18) considered traffic-related air pollution (BC, NO_x_, NO_2_, traffic noise, proximity to a major road, traffic PM), and their findings supported an association between air pollution exposure and dementia-related outcomes. Furthermore, one study supported a positive association of mild cognitive impairment (MCI) with road traffic noise [45] and a second study supports an association between shorter distance to a very busy road (10,000 cars per day) and poorer performance in a general assessment of cognition and a selective attention test [40].

Two studies used neuroimaging to show an adverse association between PM exposure and neurodegeneration (PD and white matter (WM) loss) [38,46]. One study supported an adverse association between PM and performance on a general ability test (working memory and orientation) [47], another supported an adverse association with visuo-spatial ability [41], another supported an adverse association with reasoning, short term memory, and verbal fluency [37]. Other studies identified adverse associations between cognitive decline and performance of mini mental state examination (MMSE) [42,48] and an adverse association between PM and hospitalization for neurodegenerative diseases [44]. Four studies found no association between PM exposure and the health outcomes of interest based on examining medical records or participant’s reports [43,49,50,51].

There was a wide range of techniques used to assess neurodegeneration and here we discuss the validity of different approaches. There is no single test that can provide a definitive diagnosis of Alzheimer’s or other neurodegenerative disease in life. Diagnosis considers a range of cognitive abilities and functions and there is a need to look at them longitudinally. Physical and neurological examination, mental status tests (cognitive test scores, tests within a battery), neuroimaging, and medical history may all provide an insight into dementia-related endpoints. Information from laboratory tests or a physical examination can help identify health issues that can cause symptoms of dementia. Imaging technologies have revolutionized the understanding of the structure and function of the living brain to better track and diagnose the progress of Alzheimer’s disease. Each method of detecting the dementia-related outcome has advantages and disadvantages. Cognitive test scores are informative and easy to use in epidemiology studies [52], whereas using a battery of tests offer considerable advantage over traditional measures (MMSE) “for predicting and discriminating stable from deteriorating forms of suspected dementia at an individual level” [21]. Furthermore, neuroimaging plays a crucial part in the diagnosis of dementia and provides a perception of the underlying pathologic process. Neuroimaging is important not only to exclude non-AD pathologies, but also to indicate biological markers to support the AD diagnosis [53].

Chen and co-workers [46] used a neurological examination and imaging to assess cognitive decline and Chen et al. [38] included tomography scans as an outcome measure, along with a battery of validated cognitive tests. Tomography scans and especially WM loss measurement is quantitative and specific to identify cognitive decline. Jung and colleagues used both history examination and neuroimaging [26]. However, most of the studies use a variety of different cognitive tests to measure cognitive decline [37,39,40,41,42,43,45,47,48], medical records [49,50,51], or other self-reports [29].

As mentioned above, the size of the study sample, as well as the years of follow-up period were considered in the quality rating and high number of participants, many years of follow-up, and participants that were members of a larger cohort, can upgrade the studies. Kioumourtzoglou et al. [44] had the largest sample size, which included 9,817,806 adults, both men and women. The follow up period of the study was about 10 years and the study showed positive associations with all the three outcomes (PD, AD, and dementia). Chen et al. [54] had the second largest sample size, which again included men and women, 20–85 years, and had a long follow-up period (12 years). Both studies of Palacios et al. included thousands of participants and had more than 15 years of follow-up. Weuve et al. [42] had 19,409 female participants, who were part of the Nurses’ Health Study Cognitive Cohort, and a follow up period of approximately 10 years. Ranft et al. [40], which was based on the SALIA cohort, studied proximity to a major road and had a 20 year of follow up period. Jung et al. [26] performed a study of 95,690 participants over 10 years, by using history, examination, lab tests and MRI to examine the associations between air pollution exposure and the health outcome. Studies of Kirrane et al., Tonne et al. and Power et al. [29,37,39] had adequate sample size and follow up period of 17 years, 5 years, and 10 years accordingly. Ranft et al. and Power et al. found no association with PM_10_ and the cross-sectional study of Chen & Schwarts was statistically significant only for O_3_. The remaining studies are cross-sectional studies of Tzivian et al., Chen & Schwarts, Schikowski et al. and Ailshire & Clarke, with the last study having a lost to follow-up from 1986. There is also one small case control study from Wu et al. comprised of 871 participants and only three years of follow-up. All of the other studies found statistically significant results, as mentioned above, except for three that found no statistically significant associations between particulate air pollution and PD.

Most of the reviewed studies adjusted for age, sex, smoking status, and physical activity, although several studies failed to adjust for sociodemographic factors, such as education or SES, which may potentially be confounders [26,29,43,47]. Lower education and low annual income have been associated with a greater risk for dementia [55,56,57] and it is plausible that people with low income live in areas with high levels of pollution. Although it is clear that people with cardiometabolic diseases tend to be more vulnerable to air pollution and that cardiometabolic diseases are a risk factor for cognitive dysfunction, only a few studies adjusted for such conditions [38,39,44,54]. Moreover, exposure to high levels of noise impairs cognitive abilities [54], and a large fraction of ambient pollution is traffic generated, making noise a possible confounder; only two studies adjusted for environmental noise exposure [39,45].

Bias, misclassification, and selective reporting can diminish the study quality. From our 18 studies, two mentioned selection bias [40,45], where people who were cognitively impaired were less likely to participate. Five studies [37,39,49,50,51] mentioned misclassification of personal exposure levels (exposure misclassification), because the exposures were estimated from residential address, and one study [44] mentioned outcome misclassification; the measures of the diseases were based on hospital admission records, with the possibility of misclassification of diagnosis.

Most of the studies do not report a conflict of interest and were funded by a national agency [26,29,37,38,39,41,42,43,45,46,48,49,50,51,54]. However, two publications [40,47] did not contain any conflict of interest statement.

Taken together, the results from existing epidemiological studies suggest that there is a relationship between PM exposure and cognitive decline.

### 3.2. Air Pollution and Diabetes (T2DM)

The characteristics of all the epidemiology studies which have assessed the link between PM exposure and T2DM are summarized in Table 3 and Table 4. These mostly comprise cohort studies, except for five cross-sectional studies [17,58,59,60,61]. They were conducted in North America (Canada, *n* = 2 and US, *n* = 6), Europe (UK, *n* = 1, Switzerland, *n* = 2, Denmark, *n* = 1 and Germany, *n* = 3), and Asia (Korea, *n* = 1 and China, *n* = 2). For the cohort studies, the length of the follow up ranged from 3 to 22 years.

Most studies of environmental exposure (17 out of 18) examined associations with airborne particulate matter, although 12 studies also considered NO_x_, or traffic noise and proximity to a major road, and two studies considered annual mean residential BC concentration. Exposure estimates were mostly based on air monitors at fixed locations. There was one publication that investigated occupational exposure to environmental pollution, which was amongst traffic police. This study estimated exposure from PM monitors at fixed locations in different areas with various intensities of vehicle traffic [62].

A previous comprehensive meta-analysis of 12 studies, which also reviewed the study of Brook et al. [63] has shown that ambient PM and exposure to air pollutants, such as NO_2_ and O_3_, were significantly associated with an increased risk of diabetes mortality [64]. Therefore, the findings from this meta-analysis add evidence for the adverse effect of particulate air pollution on diabetes- associated mortality and although diabetes is a risk factor for many other conditions (e.g., cardiovascular disease), this meta-analysis shows what it is already established; that higher levels of air pollutants, such as PM_10_, PM_2.5_, and NO_2_ are associated with T2DM.

There are two studies ([63,65]) in our review that examined diabetes mortality. Other studies investigated this association by assessing the incidence of T2DM from hospital admissions [66], or hospital diagnosis [67], or self-reports [20]. Diabetes status can be measured directly from biomarkers [68], such as fasting blood glucose (FBG), but also because insulin resistance (IR) and inflammation are important hallmarks of T2DM, biomarkers of inflammation, such as C-reactive protein (CRP), are also relevant. Most of the studies in this review used questionnaires and interviews, that collected information on treatment, blood glucose measurements, insulin, leptin, HbA1c, and CRP levels [69,70,71,72], but some studies carried out only blood tests of the above biomarkers [17,58,73], or used both methods [24,59,74].

In five studies [24,59,69,71,74] the outcome was measured using both questionnaires, completed by the participants and blood samples taken by them. There were two studies [66,67] based on hospital admission and diagnosis, four studies [32,58,61,73] that relied on samples taken by health specialists and one [60] based on a physical examination. Finally, there were three studies [20,70,72] that only used questionnaires, which may result in misclassification because of the use of self-reported diagnosis, and could not identify undiagnosed cases of diabetes.

In terms of study sample size and follow-up, Brook et al. [63] had the largest sample size (2.1 million adults) and a 10-year follow-up period. Pope and colleagues [65] also had a very large sample size (669,046 adults) and a 22-year follow-up. Puett and co-workers [72] had 74,412 participants and Chen et al. [66] had 62,012 participants, both with a 14-year follow-up. Five studies [24,32,67,68,69,70,71,72,74] reanalysed data from existing cohorts. Studies of Eze et al., Liu et al., Wolf et al., and O’Donovan et al. [17,58,59,60,61] are all cross-sectional studies and Liu et al. [58] and both O’Donovan et al. [61] and Wolf et al. [59] had one and three years of follow-up, respectively. All of the studies found statistically significant results, as mentioned above, except for Chen et al. [73], who found that exposure to PM_10_ was associated with an increased level of FBG in the univariable analysis, whereas the results in the multipollutant model were not significant and Puett et al. who found no strong evidence between PM and T2DM.

It is known that an unhealthy diet can lead to obesity, one of the biggest risk factors of diabetes. Moreover, some studies indicate that the effects of PM and NO_2_ are more noticeable in females [75]. Almost all the studies adjusted for BMI, sex, age, education, and physical activity. Some also adjusted for family history of diabetes [20,32]; or genes and genetic predisposition already known as risk factors of T2DM. It is hypothesised from animal studies and limited human subjects’ studies, that air pollutants can decrease the normal synthesis of insulin [62], therefore in studies investigating environmental pollution it is important to adjust for occupational exposure to vapours, gases, dusts, and fumes, that are similar to urban air pollutants; only two studies did so [60,70]. Moreover, exposure to physical agents, such as noise -both indoor and outdoor-, can also modify the levels of the insulin, therefore adjustment for noise could be important, as Eze and colleagues [17] did.

All the studies mentioned limitations that can downgrade study quality, particularly information bias. Ten studies mentioned exposure misclassification [32,58,59,60,63,67,68,69,71,74] and two outcome misclassification [70,72], due to self-reporting of diagnosis. Two studies [63,65] measured the disease outcome from a mortality database and death certificates, possibly underestimating the true prevalence of T2DM. Underestimation of the effect, because of a selected sample of study subjects, was mentioned in two studies [24,65]. All the studies declared no conflict of interest and were funded by national agencies.

Taken together the results from existing epidemiological studies suggest that there is a relationship between PM exposure and T2DM.

### 3.3. Rating the Quality of Evidence According to GRADE

GRADE considers quality, publication bias, consistency, directness, and effect size and we discuss each in turn. In this system, observational studies start as low-quality evidence, but they can be rated down in a case of high risk of publication bias. There may be some doubt when the evidence derives from many small studies and especially if these studies have been commercially funded [76]. The evidence in this review comes from large observational studies and none of the studies was funded by industry. We have found some studies with statistically significant results and other ‘null studies’ of similar size; there is little evidence of publication bias.

In the GRADE scheme, the quality of evidence decreases when essential differences occur between the populations studied, or the outcomes measured, particularly if the outcomes are indirectly related to the disease [77]. For example, in our systematic review, the population of interest in all the 37 studies was adults, but some studies were conducted in women or men only [38,39,40,41,42,49,50,67,68,69], which might influence the generalisability of the reported results, as studies of both sexes do not always examine risks separately for males and female and as the two genders may have different exposures because of different occupations for example, or because, as mentioned above, some effects are more noticeable in women. Also, there is a possibility that the desired outcome may be different from the measured outcome. The use of surrogate endpoints instead of ‘patient-important outcome of interest’ can be a source of indirectness [77]. For example, in diabetes-related studies the patient-important outcomes may be the hospital admission, diabetic symptoms, or complications because of diabetes and the surrogate outcome measures blood glucose or HbA1c concentration. In our review, there are studies that assess T2DM by looking at hospital admissions [66,67], or questionnaires [70,72], but there are also studies that measure the fasting blood glucose and other blood biomarkers [32,58,61,73]. In the dementia-related studies, the patient-important outcome may be the patient’s function and behaviour, whereas the surrogate outcomes may be measures of cognitive function. In most of our dementia studies the measurement of the health outcome was cognitive function not a patient-important outcome.

GRADE also examines the consistency of the evidence; are there conflicting results in groups between studies, for example do the majority shows associations, do the studies have similar results, or is there a lack of agreement between studies? Existing evidence from meta-analysis indicates an association between air pollutants and both T2DM [75] and cognitive decline [78]. The studies that examined neurodegeneration-related outcomes mostly reported positive associations of particulate air pollution exposure and only three studies found no association [49,51,79]. We judge there is convincing evidence for consistency in the association of particulate ambient air pollution, cognitive decline, and dementia (AD), although there are conflicting results for an association between air pollution and PD. More specifically, seven studies indicated associations between fine particulate and traffic related air pollution and cognitive decline and impairment [37,39,40,41,42,45,80], two studies identified association between air pollution and AD and dementia [48,54], two studies evaluated the association between air pollution exposure and only PD positively [29,46] and one study used a time-series-like approach to link particulate matter pollution with both PD, AD, and dementia [44]. Generally, a causal role of particulate matter can be concluded from many studies and also traffic emissions and living near busy roads can be a health threat. Increased daily levels of air pollution are related to higher hospital admissions and adverse health conditions [81,82].

Exposure to air pollution has already been suggested as a contributing factor to the increasing incidence and prevalence of diabetes [83,84] and all of the diabetes-related studies but one [72], reported positive associations between particulate air pollution exposure and diabetes-related outcomes.

It is also important to look at the effect of all possible confounding factors. In our systematic review, not all the possible confounders have been accounted for. In some cases, it is hard to exclude the possibility of social confounders. Moreover, most of the studies do not address indoor sources of air pollution, which cannot be calculated using geospatial models of air pollutants, therefore personal monitoring should take place at future studies to give us a clearer picture of the impact of different sources of pollutants. Another potential confounding variable that should be better explored in future studies is noise pollution, because of the possible association with memory loss.

Effect size, according to the GRADE approach, is based on the reported odds ratio, or relative risk or hazard ratio (OR/RR/HR) for comparison. However, it is somewhat unreliable to assess the odds ratios, due to different methodological approaches in the studies, but for most studies the observed risks were all modestly increased. There are no studies with an RR or OR of 2 or more, although most of them were statistically significant (except for [49,50,51]).

The GRADE evidence is discussed further in the following section.

## 4. Discussion

T2DM and dementia are common multi-causal conditions. An unhealthy diet, obesity, stress, culture, physical inactivity, and genetic predisposition may lead to cardio-metabolic diseases, such as T2DM [25]. Furthermore, both environmental and genetic factors play a crucial role in the aetiology of neurodegenerative diseases (e.g., AD) [26], depression, delirium, stroke, traumatic brain injury, ageing, and family history [85]. There are some familial cases (that are associated with genetic abnormalities) and some environmental factors have been identified as risk factors for neurological disease (e.g., pesticide exposure), but most of these cases are idiopathic [86].

**Figure 2:**
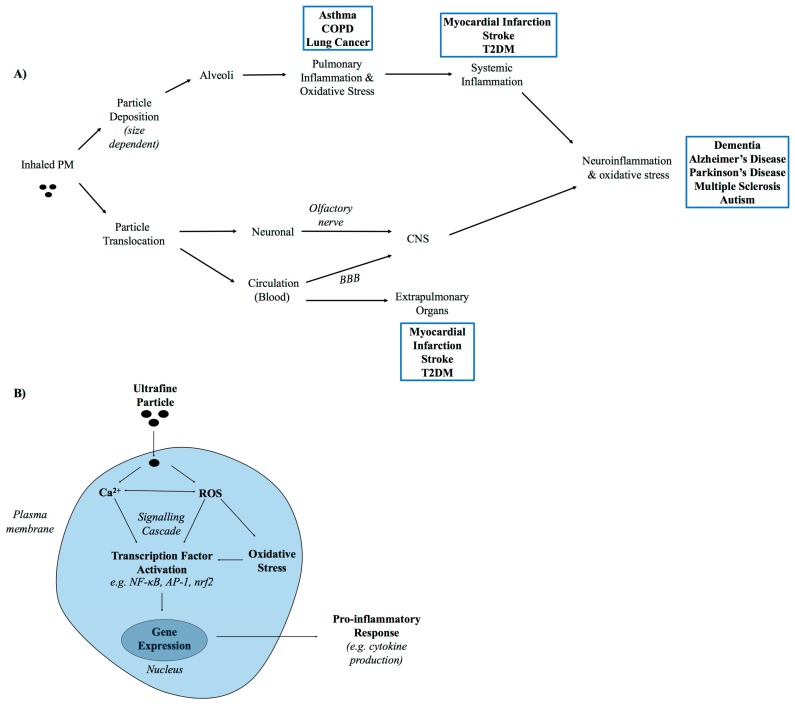
Following inhalation, particles may stimulate local or systemic effects. The hypothesised mechanism of toxicity of inhaled particles to the central nervous system (CNS) is summarized in (**A**). Impacts of inhaled particles on the CNS may emerge due to (i) particle translocation (via neurones or blood) to the CNS following inhalation or (ii) the release of systemically acting factors from the lung which impact on neurone function. Examples of the clinical impacts of inhaled particles at different target sites (lung, extrapulmonary organs, and CNS) are summarised in blue boxes; (**B**) The cellular and molecular events underlying particle toxicity to the lungs have been extensively investigated and hypothesised to involve the stimulation of inflammation and oxidative stress. More specifically, it is hypothesized that inhaled ultrafine particles interact with pulmonary cells (e.g., epithelial cells, alveolar macrophages) to stimulate an increase in intracellular ROS and Ca^2+^ concentration which leads to the expression of pro-inflammatory genes (e.g., cytokines) via the activation of transcription factors (such as NFκB). BBB = blood brain barrier. COPD = chronic obstructive pulmonary disease.

Epidemiological evidence suggests that long term exposure to particulate air pollution is a risk factor for T2DM and dementia. The mechanism underlying this is currently unknown, but it may involve the translocation of inhaled particles to the target site (e.g., CNS), or the release of (inflammatory) mediators from the lung which impact on the function of extrapulmonary organs [10,30,31] (Figure 2). In the lung, it is established that PM stimulates inflammatory and oxidative responses, which drive its toxicity (reviewed in [10,87,88]). Accordingly, both these processes are likely to be important in mediating the detrimental outcomes of PM in other organs.

More specifically, inflammation and oxidative stress are implicated in the pathogenesis of neurodegenerative diseases and T2DM. Therefore, exposure to PM may contribute to the initiation of disease pathogenesis or accelerate disease development potentiating existing responses.

Chronic inflammation has been found to be associated with the development of T2DM in humans [89]. Furthermore, a chronic inflammatory response is associated with obesity [90], one of the main risk factors for T2DM. Insulin resistance (IR) is one of the most important hallmarks in the pathogenesis of T2DM. IR is directly linked with a variety of inflammatory responses and these responses play a crucial role in the development of the condition [91]. Indeed, quantification of inflammatory mediators in blood is used as a biomarker for T2DM [92]. Individuals with T2DM have elevated cytokine levels in blood compared to people without diabetes and there is mounting evidence that supports that diabetes and particulate air pollution are associated with inflammatory dysregulation [22,93,94]. In addition, there is evidence that increased exposure to PM is significantly associated with increased fasting blood glucose (a biomarker for diabetes) in humans [73,95]. Whilst epidemiological evidence suggests that PM exposure is a risk factor for T2DM development, further research is required to better understand the cellular and molecular events underlying this as few studies have investigated the mechanism of toxicity, to date.

Numerous studies have established the role of neuroinflammation in both AD and PD pathology [28,96]. When inflammation is activated, neurones, and microglia cells release pro-inflammatory mediators (e.g., cytokines) which can stimulate an inflammatory response which damages neuronal cells and ultimately brain tissue [97,98]. The stimulation of inflammation is key to the pathogenesis of dementia. For example, the degree of inflammation correlates with brain atrophy and the severity of dementia in early AD [99]. There are also markers of inflammation, such as C-reactive protein levels (CRP) and interleukin-6 (IL-6) that are elevated in the blood plasma of patients with AD and vascular dementia [100]. An increase in the levels of the inflammatory markers in blood is associated with an increase in the risk of all types of dementia [101]. Moreover, it is already known that higher levels of inflammatory markers are associated with greater brain atrophy than expected for age [102].

Exposure to particulate air pollution, has been linked also with brain inflammation [103]. The first indications that inhaling polluted air could cause neurodegeneration came from an experiment with demented dogs in Mexico City. In this experiment, canines were exposed to significant concentrations of ozone, PM, and other pollutants. The researchers identified inflammation in the brains of the dogs along with endothelial damage, which prompted the hypothesis that the initial inflammatory source was the respiratory tract [104]. There is now evidence from several in vivo studies that PM can stimulate inflammatory and oxidative responses in the CNS [104,105,106,107,108,109,110]. Furthermore, Hullmann et al. [111] demonstrated that diesel exhaust particles accelerated the development of hallmarks of Alzheimer’s Disease in a mouse disease model. In addition, Finch and Morgan [112] showed in mice that inhalation of particulate polluted air activated the brain’s microglia, which stimulated an inflammatory response that was linked to memory loss and the pollution-exposed mice showed signs of brain damage. They suggested that the fine airborne particles might travel from the nasal cavity to the brain. Interestingly, few in vitro studies [48,109,113,114,115] have investigated the response of neurones to PM_10_ despite evidence from epidemiology and in vivo studies that PM_10_ can cause neurotoxicity.

Exposure to environmental toxicants (such as air pollutants) increases the risk of Parkinson’s disease [116] and there is evidence that neuroinflammation is the etiopathogenesis not only for Alzheimer’s disease, but also of Parkinson’s disease [28,117]. Long-term exposure to particulate air pollution may cause damage to dopaminergic neurons and lead to chronic brain inflammation to accelerate AD and PD development [29].

Epidemiological evidence has identified that diabetes and neurodegenerative diseases are linked, for example it is known that people with a metabolic syndrome, such as diabetes, are at higher risk of developing cognitive impairment [118] and Alzheimer’s disease. IR links obesity with pre-diabetes and diabetes and is associated with an increased risk for cognitive decline [119] and age-related memory impairment and AD [120]. Metabolic syndrome is considered as an independent risk factor for pre-AD syndrome and AD [121,122,123,124] and generally diabetes is a known risk factor for cognitive dysfunction and all-cause dementia [125]. T2DM is similar to AD in many ways. They are both associated with impaired glucose uptake, increased oxidative stress, inflammation, ageing, brain atrophy and they may both cause impaired cognition and dementia. This suggests that these two diseases share many factors in terms of pathophysiology and clinical outcome and due to the similarity and the pathophysiological bridge between them, AD is often referred as “type 3 diabetes” [126,127]. There is also a link between diabetes and Parkinson’s disease [128]. Individuals with T2DM are at increased risk of developing PD [129,130].

The relationship between diabetes and neurodegenerative disease may arise as a consequence of a common inflammatory mechanism [131]. For example, among individuals with metabolic syndrome, those with a higher level of inflammation are at higher risk of developing cognitive impairment compared to those with low inflammation [132]. Whilst there is evidence that inflammation is linked to both T2DM and neurodegenerative diseases, it is uncertain whether T2DM is a prerequisite to develop neurodegenerative disease from this inflammatory mechanism, or whether inflammatory processes act independently to cause these diseases. Furthermore, the hormone insulin plays an important role in memory and in brain function [120] and thus dysregulation of insulin signalling has been linked to metabolic and neurodegenerative disorders [133,134]. Accordingly, several conditions, including T2DM, activate a range of inflammatory, metabolic, and oxidative changes that might contribute to deleterious effects on the brain and other metabolic changes that might potentially drive neurodegenerative processes [135].

In this systematic review, we considered 37 studies and both levels and assessment of exposure and outcome definitions varied. The majority of the studies (31 out of 37) indicated a positive association between particulate traffic-related air pollution and cognitive impairment and/or T2DM. However, the quality of the epidemiological evidence is considered ‘poor’ because of the observational nature of the studies and there are not RCT studies that would increase the quality of the evidence. The quality of the evidence base is also poor either because it is often based on self-reports rather than on objective cognitive tests, clinical examination or neuroimaging. Furthermore, diabetes-related studies that were based on mortality data, could have underestimated the true prevalence of the disease.

Noise exposure is closely correlated with traffic related air pollution, and noise exposure is an independent risk factor for neurodegenerative diseases [136,137]. Moreover, when investigating noise or traffic proximity, which is an indicator of traffic noise, it is important to consider hearing impairment with ageing in the adult population. It is important that epidemiological studies adjust for noise exposure, although the majority of existing studies did not. It is also important that studies consider established risk factors of the disease such as obesity, nutrition, and active and passive smoking. The majority of the studies adjusted for smoking status, but only few adjusted for certain food consumption; such as fish consumption, which is associated with the risk for dementia [138]. A healthy survivor effect may also be important in some studies, because with ageing, some diabetes or dementia patients could die prematurely and no longer participate. Other lifestyle factors or other health status covariates should also be considered, such as activities of everyday life—i.e., computer experience or depression symptoms—because they play a crucial role in cognitive performance and only few studies considered them. Lack of information on these could lead to a risk of bias. Moreover, family history of cardiovascular disease can also be important and should be considered, but some studies only mentioned cardiovascular risk factors or already existing cardiovascular disease.

We were not able to perform a meta-analysis because of the variety of different outcomes that were described in the studies. However, previous review studies on T2DM have done meta-analysis for different exposure subgroups and all support an association of air pollutants with an increased risk for T2DM [18,75,139]. In practice a meta-analysis would be feasible with any of the morbidity or mortality endpoints provided there were sufficient contributing studies with consistency in the definition of the outcomes.

There are many occupations that are exposed to airborne particles, but there is only one epidemiological study that is informative about possible risks of T2DM and dementia from such exposure [62]. In workplaces, ultrafine particles are found in metal and polymer fumes and both can induce acute inflammation responses in the lung upon inhalation [140]. Many other occupational aerosol exposures can cause chronic lung inflammation—some closely related to air pollution, such as diesel engine exhaust particulate and others, such as respirable crystalline silica, that cause inflammation because of chemical or surface properties. Carbon black workers may show lung function reduction along with pro-inflammatory cytokines secretion [141]; exposure to dust can cause pulmonary reactions in dairy farmers, cotton workers, and wood workers; and inhalation of irritants can demonstrate pulmonary inflammation [142]. Also ultrafine particles can affect the nasal epithelium and produce inflammation that damages the brain [31].

As we have seen, inflammation is the key biological process linked to T2DM and neurodegeneration. By analogy, we judge that occupational particulate exposure that causes inflammation is a ‘possible’ cause of T2DM and neurodegenerative diseases due to the relatively high exposures that are likely to occur in the work environment. However, further research is needed to clarify whether there is a risk from workplace particulate exposures.

## 5. Conclusions

Despite the various studies investigating the association between air pollution and T2DM and cognitive impairment and neurodegeneration, the role of air pollution in the causation of these disorders is not fully understood and remains unclear. Available evidence indicates a positive association of ambient particulate air pollution and both T2DM and neurodegeneration risk, but corresponding evidence for similar workplace exposures is lacking. However, it is plausible that such an association between fine aerosols in the workplace are associated these diseases. Further research is required to identify the link and toxicological mechanisms associated with particulate exposure and T2DM and neurodegenerative disease. Future studies could fill key evidentiary gaps and thereby lead to additional steps to decrease air pollution levels and improve policies in the workplace environment to decrease the incidence of T2DM and cognitive decline, creating a healthier and more sustainable future.

## Figures and Tables

**Table 1:** Details of studies investigating the relationship between exposure to air pollution and cognitive function.

No.	Author’s Name & Year	Study Design/Type of Study	Population Participated Location Study Period (Average Duration of Follow-Up)	Measures of Exposure	Measures of Outcome/Disease	Confounding Factors/Adjusted for:	OR/RR/HR/β Coef (95% CI) Associations of Air Pollution with the Disease	Summary of Findings/Conclusions	Potential Bias (Limitations of Study)
1	Ailshire and Clarke, 2014 [47]	Cross-sectional from the ‘Changing Lives Study’	*N* = 780 55 years or older Men + women White + black U.S. 15 years	PM_2.5_ measured by air monitoring within 60 km of residence (data from EPA AQS)	Working memory and orientation (Serial 3 s subtraction test SPMSQ questionnaire)	Age, sex, race, education, income, employment status, residential tenure, and marital status	10 μg/m^3^ increase in PM_2.5_ associated with increased incidence rate: OR: 1.53 (1.02, 2.30).	Adverse effect of exposure to PM_2.5_ on cognitive function among older adults	Neighbourhood based measure of exposure may not fully capture individual exposure. Screening test lacks word recall tasks to assess memory. Lost to follow up from 1986 and only a selective group survived to respond. Unable to determine effects of long-term exposure. Unable to control other confounders such as diet.
2	Chen & Schwartz, 2008 [43]	Cross sectional (3rd National Health and Nutrition Examination Survey)	*N* = 1764 20–59 years U.S. 4 years	Annual home PM_10_ and O_3_ assigned to participants via geocoding (data obtained from US EPA AIRS)	Three neurobehavioral tests (SRTT, SDST, SDLT)	Age, sex, ethnicity, SES (education and employment status, annual family income, poverty-income ratio, family size), lifestyle (smoking, alcohol consumption, physical activity), urban/rural residence, cardiovascular risk factors (BMI, hypertension, diabetes mellitus, HDL). Indoor air pollutant sources.	Increase in PM_10_ by 10-μg/m^3^ associated with: SRTT (β: −0.36, −2.58 to 1.85); SDST (β: 0.00, −0.04 to 0.05); SDLT trials to criterion (β: 0.09, 0.00 to 0.17); SDLT total (β: 0.12, −0.07 to 0.31)	Adverse effects of ambient air pollutants on CNS in adults/statistically significant only O_3_ with SDST and SDLT, all the other no significant	Cross-sectional study design. The one-time residential information does not allow to characterize life-course cumulative exposure. No personal air pollution exposure monitoring data. Possibility that the observed effect of ozone may represent other photoreactive pollutants. Possibility of other confounders.
3	Chen et al., 2015 [38]	Prospective study	*N* = 1403 Women (65–80) U.S. 10 years	Spatiotemporal model (BME)-based estimated PM_2.5_ concentration	Annual screening using 3MS Examination, CERAD, tomography scans, laboratory tests	Age, race, SES, smoking, alcohol, physical activity, clinical characteristics, hypertension, diabetes, CVD	WM with fine particulate matter exposures linear regression coefficients: −5.52 ± 1.22	PM_2.5_ exposure may contribute to WM loss in older women	One-time assessment of brain volume. Not generalized findings because of sample. Only focus on PM_2.5_. Not include genetic determinants of brain structure. Only late-life exposure because of PM_2.5._
4	Chen et al., 2017 [46]	Nested case-control study (National Health Insurance Research Dataset)	*N* = 54,524 ≥40 years Taiwan 14 years	Concentrations from 76 monitoring stations across Taiwan (data from EPA of Taiwan)	Neurological examination and imaging	Age, gender, air pollution levels, urbanization levels, comorbid disease (hypertension, diabetes, dementia, stroke, depression, renal disease, sleep disorder, alcohol-related disease, head injury)	PM_10_ and PD: OR (95% CI) 1.35 (1.12, 1.62)	PM_10_ significantly affected the incidence of PD, but O_3_, CO, NO, NO_x_, NO_2_ did not	Lack of data on related biomarkers or risk factors. Diagnostic bias because of cases identified by ICD-9-CM codes. Possible attendance bias (subsequent diagnosis).
5	Chen et al., 2017 [54]	Population based cohort study	*N* = 2,165,268 20–85 years Ontario, Canada 12 years	Residential proximity to major roadways or high ways based on postal code—PM_2.5_ from a global atmospheric chemistry transport model and NO_2_ from national land-use regression model	Dementia and PD diagnoses from validated databases	Age, sex, pre-existing comorbidity (coronary heart disease, stroke, congestive heart failure, diabetes, hypertension, arrhythmia, traumatic brain injury), SES, education, income, unemployment, immigration status, urban residency	Association between major road and dementia HR (95% CI) Less than 50 m: 1.07 (1.06, 1.08) 50–100 m: 1.04 (1.02, 1.05) 101–200 m: 1.02 (1.01, 1.03) 201–300 m: 1.00 (0.99, 1.01)	Living near major roadways was associated with increased dementia incidence (but not PD or multiple sclerosis). NO_2_ and PM_2.5_ were positively associated with dementia	Did not examine factors such as noise or additional pollutants, could not identify undiagnosed cases (incomplete diagnosis might lead to underestimation of the true effect), no information on medications that may influence dementia risk, lack of information on individual SES and behavioural variables, no personal exposure assessment (assessment based on postal-code address).
6	Jung et al., 2014 [26]	Prospective Cohort study	*N* = 95,690 ≥65 Taiwan 10 years	Hourly PM_10_ and O_3_ from monitoring stations—geographic info system—spatial resolution 100.00 m (from EPA Taiwan)	From database: coding was assigned by physician (history, examination, lab, CT, MRI)	Age, sex, income, diabetes, hypertension, myocardial infarction, stroke, PAD, asthma, COPD	HRadj (95% CI) corresponding to 4.34 μg/m^3^ increase in PM_2.5_ exposure: Change CO 2.17 (2.03, 2.33), Change NO_2_ 2.23 (2.07, 2.41), Change O_3_ 2.43 (2.30, 2.57), Change SO_2_ 2.34 (2.17, 2.52)	Higher concentrations of O_3_ were associated with increased risk of newly diagnosed AD and long-term exposure to O_3_ and PM_2.5_ are associated with increased risk of AD	Not able to adjust for confounders such as genetic information, BMI, smoking, metals, occupational exposure. Did not evaluate subtypes of AD. Unable to investigate how pollutants influenced AD (no info on compositions and source of PM_2.5_).
7	Kioumourtzoglou et al., 2016 [44]	Time series analyses from Medicare open cohort	*N* = 9,817,806 Men + women More than 65 years U.S. (50 cities) 10 years	Average of all monitors for estimation of annual PM_2.5_ (data from US EPA AQS)	Admission records for PD, AD, and dementia by using codes from the ICD-9-CM	Sex, age, race, ZIP code of residence, median income, diabetes, COPD, CHF, MI	For PD: HR: 1.08 (1.04, 1.12), for AD: HR:1.15 (1.11, 1.19) and for dementia: HR:1.08 (1.05, 1.11)	Significant positive associations between long-term PM_2.5_ and PD, AD and dementia/air pollution likely accelerates the progression of neurodegeneration	Outcome misclassification (hospital admissions might be recorded with misclassifications). Mobility issues due to average age. Some subjects could have been hospitalized before turning 65.
8	Kirrane et al., 2015 [29]	Cohort (Agricultural Health Study)	*N* = 82,935 North Carolina& Iowa Min 8 years–max 17 years.	Annual averages of pollutant concentrations by using geocoded addresses 12 × 12 km grids/hierarchical Bayesian model	Self-reports of PD	Age, sex, state, race, education, smoking status, pesticide use	O_3_ and PD in NC: OR (95% CI) 1.39 (0.98, 1.98) PM_2.5_ and PD in NC: 1.34 (0.93, 1.93)	Positive associations between PD and O_3_ and PM_2.5_ concentrations in NC. In IA, associations were generally weak	Possibility of residual confounding by pesticide exposure or confounding by other occupational risk factors for PD that are different in applicators and spouses.
9	Liu et al., 2016 [51]	Nested case-control analysis based on National Institutes of Health-American Association of Retired Persons Diet and Healthy Study prospective cohort	*N* = 4869 (case: 1556 and control: 3313) U.S. 12 years	Used residential locations to estimate outdoor pollutant concentrations/daily PM_10_, PM_2.5_ and hourly NO_2_ were obtained from U.S. EPA/regionalized national universal kriging& land use regression model	Medical records and diagnostic questionnaire obtained by physician/neurologist and then reviewed by research team	Age, sex, race, smoking status, caffeine intake, physical activity, education, residential setting	PM_2.5_ and risk of PD: OR (95% CI) 1.02(0.94, 1.10) PM_10_ and risk of PD: 1.02 (0.97, 1.09) NO_2_ and PD: 1.01 (0.93, 1.10)	No statistically significant associations between exposures to ambient PM_10_, PM_2.5_, or NO_2_ and PD risk/although they found a higher risk of PD among both women and never smokers with exposures to high levels of PM_2.5_ and PM_10_	Possible misclassification. No info on concentrations in microenvironments. Pollutant estimates only in adulthood and not earlier. Only collected residential address (pollutants in workplace were not available). PD diagnosis asked only once at the follow-up survey. PD case identification based on self-reports.
10	Palacios et al., 2014 [50]	Prospective cohort	*N* = 115,767 Women U.S. 18 years (average follow up 16.6 years)	Spatio-temporal models/estimation of PM_10_ and PM_2.5_ (data from EPA’s AQS-IMPROVE)	Medical records and questionnaire from neurologist and then reviewed by movement disorder specialist	Age, region, pack years smoking, smoking status, population density, caffeine consumption, use of ibuprofen, income	PM_10_ and risk of PD: RR (95% CI) 1.03 (0.78, 1.37) PM_2.5_ and risk of PD 1.10 (0.83, 1.45)	No statistically significant associations between air pollution and PD risk	Information on air pollution from 1988 onwards (only adulthood exposure). No personal air pollution measurements (indirect measures of air pollution). Misclassification of biologically relevant levels of individual exposure. Potential occupational exposure (only info on residential address).
11	Palacios et al., 2017 [49]	Prospective cohort	*N* = 50,352 40–75 years old Male U.S. 30 years	Monthly average PM_10_ and PM_2.5_ Questionnaires using spatiotemporal models (data from EPA’s AQS)	Participant reports PD and then contact the neurologist who completes a questionnaire to confirm diagnosis and send medical record which were reviewed by a movement disorder specialist	Age, time period, smoking, region, population density	PM_10_ and PD: HRadj: 0.85 (0.63, 1.15) PM_2.5_: 0.97 (0.72, 1.32) PM_2.5–10_: 0.88 (0.64, 1.22)	No statistically significant association between PM_10_, PM_2.5_, PM_2.5–10_, and PD risk	No personal air pollution measurements, misclassification of biologically relevant levels of individual exposures, not able to account for occupational exposure to air pollution or neurotoxins, study based in U.S. only, estimate exposure only during adulthood, not generalizable results because of the sample used (highly educated male US professionals).
12	Power et al., 2011 [39]	Cohort 12 years prospective of the Normative Aging Study	*N* = 680 men only 51–97 years old Boston Massachusetts area 12 years	Black carbon from land use regression model, monitoring sites	Global cognitive functioning MMSE; digit span backwards test, verbal fluency, constructional praxis, immediate recall, delayed recall, pattern comparison task (7 cognitive tests)	Age, education, alcohol intake, physical activity, diabetes, dark fish consumption, computer experience, first language, percentage of participant’s census tract that is non-white, % of participant’s census tract with at least a college degree, cognitive data from first cognitive assessment, part time resident of greater Boston area, smoking, BMI	Doubling of black carbon concentration associated with increased risk of having a low MMSE score (ORadj: 1.3, 1.1–1.6) Doubling of black carbon concentration associated with reduced overall cognitive test score (−0.054, −0.103 to−0.006)	Significant association of higher BC with greater risk of poor cognition and worse general cognitive performance. (No association with PM_10_)/traffic related air pollution may have adverse effect on cognition in older men	Exposure estimates based on residential address may misclassify personal exposure levels. Inability to attribute findings to a particular traffic-related exposure.
13	Ranft et al., 2009 [40]	Cohort prospective (SALIA: Study on the Influence of air pollution on Lung function, Inflammation and Aging)	*N* = 402 68–79 years old Female Germany 20 years	PM_10_ by monitoring stations 8 km grid and Distance of address to next busy road with 10,000 cars per day monitoring stations by State Environment Agency	Cognitive function CERAD-Plus; Stroop test, sniffing sticks (validated)	Age, education, regular sporting activities, obesity, smoking, ETS, indoor air pollution exposure, depression, diabetes, hypertension, cholesterol, stroke, morbidity	Traffic exposure associated with CERAD test: β = −3.8 (−7.8, 0.1) Stroop: β = −5.1 (−8.2, −2.0) Sniffing: β = −1.3 (−2.4, −0.2) PM exposure associated with CERAD: 0.4 (0.0, 0.9), Stroop: −0.0 (−0.4, 0.4), Sniffing: 0.0 (−0.1, 0.1)	Significant association of shorter distance to road with worse performance on a general assessment of cognition and a test of selective attention. No association with PM_10_/chronic exposure to traffic-related PM may be involved in the development of MCI	Selection bias (due to increase of AD incidence after 74 years and disability to participate). Results are the consequence of traffic noise. Only subjects of a bigger cohort (SALIA) who were able and willing to attend follow-up 2007–2008.
14	Schikowski et al., 2015 [41]	Cross-sectional (from the SALIA cohort)	*N* = 789 Female Germany 27 years	NO_2_, NO_x_, PM_2.5_, and PM_10_ estimated using land use regression models. Daily traffic load within 100 m of residential address	Global cognition CERAD-plus, MMSE	Smoking status, ETS exposure, educational level, SES, physical activity, chronic respiratory diseases, cardiovascular diseases, body mass index, emotional state	Increased traffic load associated with CERAD: β = (−0.40; −2.16, 1.36) and MMSE (0.04; −0.18, 0.26) Increased NO_2_ associated with CERAD (−1.10; −2.37, 0.18) and MMSE (0.00; −0.16, 0.16) Increased NO_x_ with CERAD (−1.35; −2.59, −0.10) and MMSE (−0.04; −0.19, 0.12) Increased PM_10_ with CERAD (0.32; −0.68, 1.33) and MMSE (0.07; −0.06, 0.20) Increased PM_2.5_ with CERAD (0.31; −1.11, 1.72) and MMSE (0.07; −0.10, 0.25)	Markers of air pollution associated with cognitive impairment/air pollution may affect only specific areas on the brain and result in lower performance in the subtest of the CERAD test battery	Only cross-sectional analysis of air pollution exposure and cognitive function (even if applied back-extrapolation they did not know if pattern remained the same for the entire study period). Only one assessment of cognitive function at a single time point.
15	Tonne et al., 2014 [37]	Longitudinal cohort study	*N* = 2867 66 ± 6 years London, UK 5 years	Average PM_10_, PM_2.5_; average exposures from vehicle exhaust PM_10_; PM_2.5_ measured over 5 years (at 20 × 20 resolution)	Reasoning, short term memory, verbal fluency (Alice Heim 4-I Test, 20-word free recall, semantic and phonemic verbal fluency)	Age, sex, ethnicity, marital status, educational achievement, socioeconomic position, smoking status, alcohol use, frequency of fruit and vegetable consumption, physical activity, systolic and diastolic blood pressure, serum cholesterol levels, prevalence of stroke, coronary heart disease and diabetes, frequency of depressive symptoms, year of screening	Higher PM_2.5_ of 1.1 μg/m^3^ was associated with a 0.03 (95% CI 0.002–0.06) 5-year decline in standardized memory score and a 0.04 (−0.07–0.01) decline when restricted to participants remaining in London between study waves	Association between PM and reasoning and decline over time in memory, no conclusive findings for verbal fluency	Exposure misclassification (exposure was based only at residence (not take into account workplace etc) and the role of air conditioning). No data on traffic noise exposure (confounder). Only two cognitive assessments.
16	Tzivian et al., 2016 [45]	Cross-sectional (based on Heihz Nixdorf Recall study)	*N* = 2050 45–75 years old German Ruhr Area (3 cities) 5 years	PM was measured in 20 sites, NO_x_ was measured at 40 sites over 1 year—noise exposure assessment (land use regression)	Verbal memory, speed of processing, verbal fluency, abstraction (MCI diagnosed according to Petersen/International working group on MCI criteria)	Age, sex, SES, alcohol consumption, smoking status, ETS, physical activity, BMI, CHD, T2DM, APOEε4, depression	PM_10_ OR (95% CI): 1.11 (0.99, 1.23) PM_2.5_: 1.16 (1.05, 1.27) NO_x_: 1.10 (0.96, 1.26) Traffic noise: Lden: 1.40 (1.03, 1.91) Lnight: 1.80 (1.07, 3.04)	Long-term exposure to both air pollution and road traffic noise was associated with overall MCI-strongest associations for PM_2.5_	Cross-sectional design. Selection bias (cognitively impaired people less likely to participate). Underreporting (questionnaires). Possible exposure misclassification and residual confounding between air pollution and noise.
17	Weuve et al., 2012 [42]	Prospective (Nurses’ Health Study Cognitive Cohort)	*N* = 19 409 Age 70–81 years Female U.S. 7–13 years	Quintiles of PM_2.5_ and PM_2.5–10_ in preceding month, year, 2 years, 5 years, and since 1988 (monitor data obtained from USEPA AQS)	Cognitive functioning TICS, East Boston Memory Test (immediate and delayed paragraph recall)	Age, education, husband’s education, physical activity, smoking status, alcohol consumption, history of diabetes, coronary diseases, high blood pressure, emphysema	PM_2.5_ highest vs. lowest quintile of long-term exposure associated with greater 2-year decline in global cognition (−0.018; 95% CI: −0.034, −0.002) PM_2.5–10_ highest vs. lowest quintile of long-term exposure associated with greater 2-year decline in global cognition (−0.024; 95% CI: −0.040, −0.008)	Higher levels of exposures to ambient PM are associated with worse cognitive decline	Indirect estimates of PM results due to confounding.
18	Wu et al., 2015 [48]	Case-control study	*N* = 871 ≥60 years old Taiwan 3 years	Estimation of spatiotemporal distribution of PM_10_ (and ozone) concentration (data from EPA Taiwan)	Mini mental state examination (Diagnostic and Statistical Manual of Mental Disorders)	For AD: age, gender, APOE ε4 status, PM_10_ level, ozone level, education years, BMI	Association of PM_10_ and risk of dementia: OR (95% CI) 4.17 (2.31, 7.54) *p* < 0.0001	Elevated long-term PM_10_ level was significantly associated with an increased risk of AD and VaD in the elderly	Explored only two air pollutants. Assumption that participants tended to live in the same places after retirement. Survival bias (people who did not survive for 12 to 14 years).

EPA AQS = Environmental Protection Agency’s Air Quality System, SPMSQ = Short Portable Mental Status Questionnaire, AIRS = Aerometric Information Retrieval System, SRTT = Simple Reaction Time Test, SDST = Symbol-Digit Substitution Test, SDLT = Serial-Digit Learning Test, CERAD = Consortium to Establish a Registry for Alzheimer’s Disease, MMSE = Mini Mental State Examination, ETS = Environmental Tobacco Smoke, MCI = Mild Cognitive Impairment, CHD = Coronary Heart Disease, USEPA = US Environmental Protection Agency.

**Table 2:** Summaries of exposure and dementia-related outcome considered.

No.	Author’s Name and Year	Exposure/Pollutants				Outcomes					
		PM_10_	PM_2.5_	NO_x_/NO_2_	BC and Others (such as O_3_, CO, SO_2_)	Cognitive Decline	MCI	A.D.	P.D.	Dementia	Neurodegeneration
1	Ailshire and Clarke, 2014 [47]		×			×					
2	Chen & Schwartz, 2008 [43]	×			×	×					
3	Chen et al., 2015 [38]		×								× (WM loss)
4	Chen et al., 2017 [46]	×		×	×				×		
5	Chen et al., 2017 [54]		×	×					×	×	
6	Jung et al., 2014 [26]		×		×			×			
7	Kioumourtzoglou et al., 2016 [44]		×					×	×	×	
8	Kirrane et al., 2015 [29]		×		×		×				
9	Liu et al., 2016 [51]	×	×						×		
10	Palacios et al., 2014 [50]	×	×						×		
11	Palacios et al., 2017 [49]	×	×						×		
12	Power et al., 2011 [39]				x	×					
13	Ranft et al., 2009 [40]	×			×		×				
14	Schikowski et al., 2015 [41]	×	×	×		×					
15	Tonne et al., 2014 [37]	×	×			×					
16	Tzivian et al., 2016 [45]	×	×	×	×		×				
17	Weuve et al., 2012 [42]	×	×			×					
18	Wu et al., 2015 [48]	×								×	

**Table 3:** Details of studies investigating the relationship between exposure to air pollution and diabetes mellitus.

No.	Author’s Name & Year	Study Design/Type of Study	Location/Population Participated Study Period (Average Duration of Follow-Up)	Measures of Exposure	Measures of Outcome/Disease	Confounding Factors/Adjusted for:	OR/RR/HR/β Coef (95%CI) Associations of Air Pollution with the Disease	Summary of Findings/Conclusions	Potential Bias (Limitations of Study)
1	Brook et al., 2013 [63]	Prospective cohort	*N* = 2.1 million adults Canada 10 years	Average concentrations of PM_2.5_ from satellite data with a spatial resolution of 10 × 10 km	Diabetes mortality from Canadian Mortality Database	Sex, age, any aboriginal ancestry, marital status, education level, employment status, occupation classification, income	HR (95% CI) stratified by age & sex: 1.10 (1.03, 1.18) Measured at individual level: 1.30 (1.21, 1.39) Including community size: 1.51 (1.39, 1.64) Other contextual variables: 1.49 (1.37, 1.62)	PM_2.5_ was significantly associated with diabetes mortality	Cross-coding and misclassification because underlying cause of death may be difficult to establish. Underestimation of true prevalence of diabetes because of use of death certificates. Possibility of confounding by regional differences in coding. Diabetes-related deaths were not capture in this study. Exposure misclassification.
2	Chen et al., 2013 [66]	Population-based cohort	*N* = 62,012 ≥equal to 35 years (mean age: 54.9) Ontario, Canada 14 years max (mean follow up: 8 ± 3.2 years)	Satellite-based estimates of surface concentrations of PM_2.5_ (NASA’’ satellite) at a resolution of approximately 10 × 10 km	Enter Diabetes database if at least one hospital admission with diabetes diagnosis or 2 or more physicians claims for diabetes (2 year period)	Marital status, race/ethnicity, education, household income, BMI, smoking status, alcohol consumption, daily consumption of fruits and vegetables, physical activity, urban/rural residence, hypertension, area-level unemployment, COPD, heart failure, acute myocardial infarction, asthma	For a 10 μg/m^3^ increase in PM_2.5_ HRadj (95% CI): 1.11 (1.02, 1.21)	Long-term exposure to PM_2.5_ was associated with an increased risk of incidence diabetes after controlling for various individual and neighbourhood covariates	Not differentiate between type 1 and 2 diabetes. Could not identify undiagnosed cases of diabetes in cohort. Unable to estimate associations at finer spatial scale. No info on daily activity. Do not consider the mixture of air pollutants. No family history of diabetes or occupational exposure.
3	Chen et al., 2016 [73]	Prospective population-based cohort (Kailuan cohort)	*N* = 27,685 (18 to 90 years Mean of 47 years) Tangshan City, China 5 years	PM_10_ and NO_2_ obtained from Tangshan Environmental Monitoring Centre	Fasting blood samples were assayed for concentrations of glucose etc. by specialist	Age, sex, BMI, drinking status, smoking status, annual family income, education, BP, history of diabetes and hypertension and stroke, exercise activity, marital status, work type, seasonality	Univariate PM_10_ *p* value< 0.001 Multipollutant model (SO_2_ + NO_2_ + PM_10_) PM_10_ −0.047 (−0.11, 0.01) *p* value 0.094	Exposure to PM_10_ (and NO_2_ and SO_2_) was associated with an increased level of FBG/univariate analysis significant results, whereas multipollutant model was not significant	Ozone and PM_10_ not assessed. Used fixed monitoring data rather than personal air pollution exposure. Sex distribution not balanced.
4	Chen et al., 2016 [69]	Cohort	*N* = 1023 (17.9–65.6 years Mean age 34.5) Mexican American women Los Angeles, California, U.S. 7 years	PM_2.5_ and NO_2_ data collected spatial interpolation of data from air quality monitors (FRM)/ambient info from U.S. Environmental Protection Agency’s Air Quality System data max interpolation radius of 50 km	DXA and oral and intravenous glucose tolerance test (FSIGT) and completed dietary and physical activity questionnaires	SES, income, poverty rate, unemployment rate, education, physical activity, and dietary intakes	Between PM_2.5_ and fasting glucose: β(*p*): 0.08 (<0.001) HOMA-IR: 5.81 (0.016)	Higher annual average PM_2.5_ exposure was significantly associated with higher fasting glucose, HOMA-IR, and lower insulin resistance	Limitation on generalizability of our results (only overweight Mexican American). Nondifferential misclassification (personal air pollution exposure levels were not monitored). Individual-level info on SES was not available. No info on covariates of interest such as sleep, noise, smoking, and indoor sources of air pollution.
5	Coogan et al., 2012 [20]	Prospective Cohort (Black Women’s Health Study)	*N* = 4204 (21–69 years) Women Los Angeles, U.S. 10 years	PM_2.5_ and NO_x_-Participants’ residential address with land use regression models and interpolation from monitoring station measurements	A self-report of doctor diagnosed DM (then physicians provided data from their medical records)	Age, height, weight, smoking and alcohol consumption, household income, family size, education, neighbourhood SES, physical exercise	The IRRs for diabetes mellitus were 1.63 (95% CI, 0.78, 3.44) and 1.25 (95% CI, 1.07, 1.46)	Exposure to air pollutants may increase the risk of T2DM	Not feasible to identify undiagnosed cases of diabetes in the cohort. Pollutant exposures were assessed for only 1 year and assigned to all years of follow-up. Only residential address (not work address).
6	Donovan et al., 2017 [61]	Cross-sectional (CHAMPIONS study)	*N* = 10,443 (40–75 white European 25–75 other) UK 3 years	1 × 1 km grids of pollutant concentrations from DEFRA	Oral glucose tolerance test based on WHO 2011 criteria	Age, sex, smoking habit, urban or rural location, area social deprivation score, ethnicity, cholesterol, physical activity, neighbourhood green space	OR for T2DM was 1.10 (0.92, 1.32) after adjustment for lifestyle factors and 0.91 (0.72, 1.16) after further adjustment for neighbourhood green space	PM and NO_2_ were associated with T2DM in unadjusted models, no associations after certain adjustments	Causal relationships cannot be inferred because of study design. Exposure to air pollution based on residential location (may not reflect actual exposure). Associations not adjusted for confounders such as noise. Possibility of over-adjustment, bias due to missing data.
7	Eze et al., 2014 [17]	Cross-sectional of the cohort (SAPALDIA)	*N* = 6392 29–73 years Switzerland 11 years	PM_10_ and NO_2_ Validated dispersion models of 200 × 200 m resolution/Annual trends at fixed monitoring sites and participant residential histories were used to estimate residential levels	Health examinations (computer-assisted interviews, lung function, allergy testing), blood samples taken	Age, sex, BMI, education, neighbourhood SES, physical activity, smoking, alcohol, occupational exposure, raw vegetables consumption, co-morbidities (COPD), road traffic noise exposure	Fully adjusted OR for prevalent diabetes was 1.40 (95% CI: 1.17, 1.67) Unadjusted: 1.46 (1.20, 1.77)	Long-term exposure to PM_10_ and NO_2_ were positively associated with prevalent diabetes mellitus	The inclusion of all cases of self-reported, physician diagnosed diabetes irrespective of the time of diagnosis. Potential bias due to differential non-participation.
8	Eze et al., 2015 [60]	Cross-sectional (SAPALDIA)	*N* = 3769 29–73 years Switzerland 10 years	Estimates of PM_10_ and NO_2_ dispersion models (200 × 200 m)/land use regression	Physical examination	Sex, age, smoking status, physical activity, SES, occupational status of household head, alcohol intake, educational level, consumption of raw vegetables, fruits, occupational exposures to vapours/dust/fumes	Association between PM_10_ and MetS: OR (95% CI): 1.64 (1.35, 1.98) 1.58 (1.29, 1.95) 1.72 (1.46, 2.02) (3 different models)	Strongest association with MetS and PM_10_ (than NO_2_)/ positive associations between markers of long-term AP exposure and MetS	Cross-sectional design. No estimates of indoor or occupational air pollution for our participants. Physical activity not objectively measured.
9	Hansen et al., 2016 [67]	Cohort (Danish Nurse Cohort)	*N* = 28,731 44–95 years Female Denmark 20 years (mean follow up 15.3 years)	PM_2.5_, PM_10_, NO_2_ and NO_x_ concentrations air pollution dispersion modelling system	Hospital diagnosis-5 blood glucose measurements within a year—second purchase of insulin or oral anti-diabetic drugs	Age, BMI, neighbourhood SES, physical activity, smoking, alcohol, consumption of fruit and vegetables, employment status, marital status, MI, hypertension	HR for PM_2.5_ and diabetes 1.14 (1.04, 1.24) 1.11 (1.02, 1.22) 1.11 (1.01, 1.22) (3 different models)	- Long-term exposure to PM_2.5_ was associated with increased risk for diabetes - Weak positive insignificant associations between diabetes incidence and PM_10_, NO_2_, NO_x_	Exposure misclassification. Lack of info on indoor exposures-air pollution at work-commuting habits-personal activity patterns. Lack of noise exposure data. Not distinguish type 1 from type 2 diabetes.
10	Kim et al., 2012 [71]	Longitudinal study (Korean Elderly Environmental Panel)	*N* = 560 ≥60 years Seoul, Korea 3 years	PM_10_ and NO_2_ were obtained from ROK (concentrations nearest to the residence of each subject were used to estimate individual exposures, average distance monitor and residence <1 km)	Medical examinations, fasting blood samples, questionnaire about demographics, lifestyle habits and medical history (measure fasting glucose—hexokinase method and insulin levels—double antibody batch method and HOMA)	Age, BMI, sex, cotinine level, outdoor temperature, dew point	PM_10_ and HOMA: 0.14 (−0.003, 0.29)	Positive associations of PM_10_, O_3_, NO_2_ with fasting glucose, insulin, and HOMA indices, indicating that these pollutants may affect the development of DM	Results not generalizable to younger people. No measurement of individual exposure. Exposure misclassification. No SES adjustment.
11	Kramer et al., 2010 [70]	Cohort (SALIA: Study on the Influence of Air Pollution on Lung, Inflammation and Aging)	*N* = 1775 54–55 years old women Germany 16 years	PM and NO_2_-Data from monitoring stations (State Environment Agency) in an 8 km grid, and emission inventories to assess motor vehicle exhaust, land use regression models, baseline investigation to next major road	Questionnaire (physician diagnosis of diabetes, antidiabetic treatment) and interview	Age, BMI, SES, education, smoking, workplace exposure, hypertension	Adjusted HR (95% CI) Monitoring stations: PM_10_ 1.16 (0.81, 1.65) NO_2_ 1.34 (1.02, 1.76) Emission inventory: PM 1.15 (1.04, 1.27), NO_2_ 1.15 (1.04, 1.27) Land-use regression model: NO_2_ 1.42 (1.16–1.73)	- Traffic-related air pollution is associated with increased risk to develop T2DM - Stronger associations with NO_2_ than PM-related exposure assessments	Self-report only. Outcome misclassification—under diagnosis (no glucose measurements). Not complete follow-up and higher education overrepresented.
12	Liu et al., 2016 [58]	Cross- sectional study (China Health and Retirement Longitudinal Study)	*N* = 11,847 ≥45 years China 1 year	PM_2.5_-Satellite-based spatial statistical model 10 × 10 km resolution	Blood test HbA1c: Boronate affinity HPLC method Glucose levels: enzymatic colorimetric method	Age, sex, BMI, educational status, location of residence, smoking status, drinking, indoor air pollution, ambient O_3_	PRadj (95% CI) of T2DM associated with PM_2.5_: 1.14 (1.08, 1.20) Fasting glucose: 0.26 (0.20, 0.32) HbA1c: 0.08 (0.06, 0.10)	Long-term exposure to PM_2.5_ was positively associated with significant increases in diabetes prevalence, fasting glucose, and HbA1c levels	Not completely exclude exposure measurement errors cause spatial resolution of PM_2.5_ was still not very high. Did not have long-term PM_2.5_ measurements before survey for several years. Failed to have info about how long they had T2DM. Unable to control physical activity confounding. Not able to evaluate medication as possible effect modifier. Uncertainty to exposure assessment because of change in address.
13	Park et al., 2015 [32]	Prospective Cohort (Multi-Ethnic Study of Atherosclerosis)	*N* = 5135 45–84 years U.S. 12 years max (median 9 years follow-up)	PM_2.5_ and NO_x_ concentrations hierarchical spatiotemporal model (US Environmental Protection Agency’s Air Quality System)	Fasting serum glucose levels measurements	Age, sex, race, family history of DM, educational level, smoking, alcohol consumption, physical activity, NSES index, BMI, site	PM_2.5_ and DM: ORadj (95% CI): 1.09 (1.0, 1.1)	Long-term exposures to PM_2.5_ and nitrogen oxides estimated as the annual averages were significantly associated with prevalent DM at baseline (not incidence)	Exposure measures were based on annual averages from year 2000 and assumed that the exposures were time constant.
14	Pope et al., 2015 [65]	Cohort	*N* = 66,046 U.S. 22 years	PM_2.5_-Land use regression and BME interpolation model	Deaths linked to diabetes death/certificates	BMI, smoking habits, occupational exposures, marital status, education, alcohol	Per 10 μg/m^3^ increment in PM_2.5_ and diabetes mellitus: HR (95% CI): 1.13 (1.02, 1.26)	PM_2.5_ is associated with diabetes mellitus mortality	Not random sample (included friends and family members). Underestimation of the effect. Reduce precision of control for risk factors. Use of cause-of-death info.
15	Puett et al., 2011 [72]	Two prospective cohorts (Nurses’ Health Study & Health Professionals Follow-up Study)	74,412 women and 15,048 men U.S. 14 years	PM questionnaires to geocoded address and spatiotemporal models developed/using monitoring data (from US EPA AQS, VIEWS, IMROVE, CASTNet)	Reported diagnosis of DM on questionnaire	Age, season, calendar year, state of residence, time-varying cigarette smoking, hypertension, BMI, alcohol intake, physical activity, diet	HR (95% CI): 1.03 (0.96, 1.10) for PM_2.5_, 1.04 (0.99, 1.09) for PM_10_, 1.04 (0.99, 1.09) for PM_10–2.5_	No strong evidence for an association between exposure to PM_2.5_, PM_10_, or PM _10–2.5_ in the 12 months before diagnosis and T2DM incidence	Misclassification because of self-reported diagnosis. Meta-analyses and combined analyses were dominated by the NHS because of number of participants. Need of more acute exposures and exposures during childhood. No generalizability of results (narrow range of SES).
16	Wang et al., 2014 [74]	Prospective cohort (MOBILIZE Boston Study)	*N* = 765 ≥65 years mean age: 78.1 Boston, U.S. 1 year	PM_2.5_-ArcGIS spatial-temporal land-use regression model Euclidean distance from residence to nearest major roadway	Interview/clinic examination (blood samples)	Age, sex, race, season, physical activity, alcohol consumption, smoking, household income, education, neighbourhood SES, BMI, diabetes, hypertension, hyperlipidaemia	Fully adjusted model: 0.12 (0.03, 0.22) leptin levels associated with increase in BC	Evidence that leptin was associated with annual mean residential BC, but not residential distance to major road/long-term exposure to at least some aspects of traffic pollution may adversely impact cardiometabolic health	Measured leptin in non-fasting serum samples (do not know when participants last ate), measure leptin not with conventional ELISA. No info about residential history prior to enrolment. Only one leptin measurement. Exposure misclassification or residual confounding (no info about indoor home or combustion-derived pollution). No generalizable results.
17	Weinmayr et al., 2015 [24]	Cohort (Heinz Nixdorf Recall Study)	*N* = 3607 45–75 years old Germany Mean follow-up 5.1 years	PM_10_ and PM_2.5_ chemistry transport model (EURAD-CTM) on a spatial resolution of 1 km^2^ grid cells	Questionnaire, face to face interviews, clinical and lab tests, clinical examination, glucose measurements	Sex, age, BMI, smoking status, physical activity, area-level and individual-level SES, and city	Association of total and traffic-specific pollutants and diabetes incidence: RR (95% CI) Total PM_10_: 1.05 (1.00, 1.10) Total PM_2.5_: 1.03 (0.95, 1.12) Traffic PM_10_: 1.36 (0.98, 1.89) Traffic PM_2.5_: 1.36 (0.97, 1.89)	Possible effect of total PM on type 2 diabetes risk/clear effect for living near a busy road/long-term exposure to total PM increases type 2 diabetes risk in the general population	The availability of only modelled values. Could not account for the mobility of study participants. Underestimation of real risk if air pollution higher.
18	Wolf et al., 2017 [59]	Cross-sectional (KORA: Cooperative Health Research in the Region Augsburg)	*N* = 2944 mean age: 56.2 Germany 3 years	PM_10_, PM_2.5_, NO_2_, and NO_x_ monitoring sites land use regression	HOMA-IR, glucose, insulin, HbA1c, leptin, C-reactive protein from fasting samples/interview, questionnaires	Sex, age, BMI, smoking status, physical activity, waist-to-hip ratio, month of blood withdrawal, SES, per capita income, years of education, occupational status, alcohol intake	7.9 μg/m^3^ increment in PM_10_ was associated with higher HOMA-IR change (95% CI) 0.16 (0.04, 0.29) and insulin 0.15 (0.36, 0.27)	Positive associations between PM_10_, PM_2.5_, NO_2_, and NO_x_ and HOMA-IR and insulin levels/association between traffic-related air pollution and biomarkers related to IR, subclinical inflammation and adipokines in the general population	One-time measurements because of cross-sectional study design. Not possible to infer causation (biomarkers determined up to 3 years before air pollution measurements). Exposure misclassification.
Details of a study investigating the relationship between occupational exposure to air pollution and diabetes mellitus									
19	De Sio et al., 2005 [62]	Case-control	*N* = 488 Rome, Italy 2 months (March–April 2001)	PM_10_ in fixed stations located in districts with different intensities of vehicle traffic	Sample of venous blood/measure the insulin concentration using radio-immunoassay	Early risk factor for diabetes or for reduced glucose tolerance→cumulative effect of urban pollutants	In male traffic police mean plasma insulin levels were significantly lower compared with controls (*p* = 0.000). In female were also significantly lower (*p* = 0.002).	Plasma insulin level was altered in traffic police who are exposed to chemical and physical stressors	Not mentioned.

FRM = Federal Reference Method, BME = Bayesian Maximum Entropy, U.S.EPA = U.S. Environmental Protection Agency, AQS = Air Quality System, VIEWS = Visibility Information Exchange Web System, IMPROVE = Interagency Monitoring of Protected Visual Environments, CASTNet = Clean Air Status and Trends networks, NSES = neighbourhood socioeconomic status, EURAD-CTM = European Air Pollution Dispersion and Chemistry Transport Model.

**Table 4:** Summaries of exposure considered in diabetes studies.

No.	Author’s Name and Year	Exposure/Pollutants			
		PM_10_	PM_2.5_	NO_x_/NO_2_	BC and Others (such as O_3_, CO, SO_2_)
1	Brook et al., 2013 [63]		×		
2	Chen et al., 2013 [66]		×		
3	Chen et al., 2016 [73]	×		×	×
4	Chen et al., 2016 [69]		×	×	×
5	Coogan et al., 2012 [20]		×	×	×
6	De Sio et al., 2005 [62]	×	×		
7	Donovan et al., 2017 [61]	×	×	×	
8	Eze et al., 2014 [17]	×		×	
9	Eze et al., 2015 [60]	×		×	
10	Hansen et al., 2016 [67]	×	×	×	
11	Κim et al., 2012 [71]	×		×	×
12	Kramer et al., 2010 [70]	×	×	×	×
13	Liu et al., 2016 [58]		×		
14	Park et al., 2015 [32]		×	×	
15	Pope et al., 2015 [65]		×		
16	Puett et al., 2011 [72]	×	×		
17	Wang et al., 2014 [74]				×
18	Weinmayr et al., 2015 [24]	×	×		×
19	Wolf et al., 2017 [59]	×	×	×	

## Appendix A

**Table A1:** PRISMA checklist.

Section/Topic	#	Checklist Item	Reported on Page #
TITLE			
Title	1	Identify the report as a systematic review, meta-analysis, or both.	1
ABSTRACT			
Structured summary	2	Provide a structured summary including, as applicable: background; objectives; data sources; study eligibility criteria, participants, and interventions; study appraisal and synthesis methods; results; limitations; conclusions and implications of key findings; systematic review registration number.	1
INTRODUCTION			
Rationale	3	Describe the rationale for the review in the context of what is already known.	1–2
Objectives	4	Provide an explicit statement of questions being addressed with reference to participants, interventions, comparisons, outcomes, and study design (PICOS).	1–2
METHODS			
Protocol and registration	5	Indicate if a review protocol exists, if and where it can be accessed (e.g., Web address), and, if available, provide registration information including registration number.	-
Eligibility criteria	6	Specify study characteristics (e.g., PICOS, length of follow-up) and report characteristics (e.g., years considered, language, publication status) used as criteria for eligibility, giving rationale.	3
Information sources	7	Describe all information sources (e.g., databases with dates of coverage, contact with study authors to identify additional studies) in the search and date last searched.	3
Search	8	Present full electronic search strategy for at least one database, including any limits used, such that it could be repeated.	3
Study selection	9	State the process for selecting studies (i.e., screening, eligibility, included in systematic review, and, if applicable, included in the meta-analysis).	3
Data collection process	10	Describe method of data extraction from reports (e.g., piloted forms, independently, in duplicate) and any processes for obtaining and confirming data from investigators.	3
Data items	11	List and define all variables for which data were sought (e.g., PICOS, funding sources) and any assumptions and simplifications made.	8–25
Risk of bias in individual studies	12	Describe methods used for assessing risk of bias of individual studies (including specification of whether this was done at the study or outcome level), and how this information is to be used in any data synthesis.	4–5
Summary measures	13	State the principal summary measures (e.g., risk ratio, difference in means).	8–15, 17–24
Synthesis of results	14	Describe the methods of handling data and combining results of studies, if done, including measures of consistency (e.g., I^2^) for each meta-analysis.	4–5
Risk of bias across studies	15	Specify any assessment of risk of bias that may affect the cumulative evidence (e.g., publication bias, selective reporting within studies).	5, 7, 8–15, 17–24, 26
Additional analyses	16	Describe methods of additional analyses (e.g., sensitivity or subgroup analyses, meta-regression), if done, indicating which were pre-specified.	-
RESULTS			
Study selection	17	Give numbers of studies screened, assessed for eligibility, and included in the review, with reasons for exclusions at each stage, ideally with a flow diagram.	4
Study characteristics	18	For each study, present characteristics for which data were extracted (e.g., study size, PICOS, follow-up period) and provide the citations.	8–25
Risk of bias within studies	19	Present data on risk of bias of each study and, if available, any outcome level assessment (see Item 12).	8–15, 17–24
Results of individual studies	20	For all outcomes considered (benefits or harms), present, for each study: (a) simple summary data for each intervention group (b) effect estimates and confidence intervals, ideally with a forest plot.	8–15, 17–24
Synthesis of results	21	Present results of each meta-analysis done, including confidence intervals and measures of consistency.	-
Risk of bias across studies	22	Present results of any assessment of risk of bias across studies (see Item 15).	7, 26
Additional analysis	23	Give results of additional analyses, if done (e.g., sensitivity or subgroup analyses, meta-regression (see Item 16)).	-
DISCUSSION			
Summary of evidence	24	Summarize the main findings including the strength of evidence for each main outcome; consider their relevance to key groups (e.g., healthcare providers, users, and policy makers).	8–25, 30
Limitations	25	Discuss limitations at study and outcome level (e.g., risk of bias), and at review-level (e.g., incomplete retrieval of identified research, reporting bias).	30
Conclusions	26	Provide a general interpretation of the results in the context of other evidence, and implications for future research.	31
FUNDING			
Funding	27	Describe sources of funding for the systematic review and other support (e.g., supply of data); role of funders for the systematic review.	31

## References

[1] Nenonen N., Saarela K.L., Takala J., Hamalainen P. (2014). Global Estimates of Occupational Accidents and Fatal Work-Related Diseases.

[2] Health and Safety Executive (2016). Health and Safety Statistics for UK Report.

[3] World Health Organization (WHO) (2016). Ambient Air Pollution: A Global Assessment of Exposure and Burden of Disease.

[4] WHO (2009). Global Health Risks: Mortality and Burden of Diseases Attributable to Selected Major Risks.

[5] (2016). Every Breath We Take: The Lifelong Impact of Air Pollution.

[6] Donaldson K., Seaton A. (2012). A short history of the toxicology of inhaled particles. Part. Fibre Toxicol..

[7] Jun K., Satoh T. (2009). Case Study of Air Pollution Episodes in Meuse Valley of Belgium, Donora of Pennsylvania, and London, UK. Environmental Toxicology and Human Health.

[8] Seaton A., MacNee W., Donaldson K., Godden D. (1995). Particulate air pollution and acute health effects. Lancet.

[9] Donaldson K., Stone V. (2003). Current hypotheses on the mechanisms of toxicity of ultrafine particles. Annali Dell’istituto Superiore di Sanita.

[10] Stone V., Miller M.R., Clift M.J.D., Elder A., Mills N.L., Moller P., Schins R.P.F., Vogel U., Kreyling W.G., Alstrup Jensen K. (2017). Nanomaterials Versus Ambient Ultrafine Particles: An Opportunity to Exchange Toxicology Knowledge. Environ. Health Perspect..

[11] Brook R.D., Rajagopalan S., Pope C.A., Brook J.R., Bhatnagar A., Diez-Roux A.V., Holguin F., Hong Y., Luepker R.V., Mittleman M.A. (2010). Particulate matter air pollution and cardiovascular disease: An update to the scientific statement from the American Heart Association. Circulation.

[12] Dockery D.W., Pope C.A., Xu X., Spengler J.D., Ware J.H., Fay M.E., Ferris B.G., Speizer F.E. (1993). An association between air pollution and mortality in six U.S. cities. N. Engl. J. Med..

[13] Schwartz J. (1994). Air pollution and daily mortality: A review and meta analysis. Environ. Res..

[14] D’Amato G. (1999). Outdoor air pollution in urban areas and allergic respiratory diseases. Monaldi Arch. Chest Dis..

[15] Jovanovic-Andersen Z. (2012). Health effects of long-term exposure to air pollution: An overview of major respiratory and cardiovascular diseases and diabetes. Chem. Ind. Chem. Eng. Quart..

[16] WHO (2013). Air Pollution and Cancer.

[17] Eze I.C., Schaffner E., Fischer E., Schikowski T., Adam M., Imboden M., Tsai M., Carballo D., von Eckardstein A., Kunzli N. (2014). Long-term air pollution exposure and diabetes in a population-based Swiss cohort. Environ. Int..

[18] Wang B., Xu D., Jing Z., Liu D., Yan S., Wang Y. (2014). Effect of long-term exposure to air pollution on type 2 diabetes mellitus risk: A systemic review and meta-analysis of cohort studies. Eur. J. Endocrinol..

[19] Teichert T., Vossoughi M., Vierkotter A., Sugiri D., Schikowski T., Schulte T., Roden M., Luckhaus C., Herder C., Kramer U. (2013). Association between traffic-related air pollution, subclinical inflammation and impaired glucose metabolism: Results from the SALIA study. PLoS ONE.

[20] Coogan P.F., White L.F., Jerrett M., Brook R.D., Su J.G., Seto E., Burnett R., Palmer J.R., Rosenberg L. (2012). Air pollution and incidence of hypertension and diabetes mellitus in black women living in Los Angeles. Circulation.

[21] De Jager C., Blackwell A., Budge M., Sahakian B. (2005). Predicting cognitive decline in healthy adults. Am. J. Geriatr. Psychiatry.

[22] Liu C., Bai Y., Xu X., Sun L., Wang A., Wang T.Y., Maurya S.K., Periasamy M., Morishita M., Harkema J. (2014). Exaggerated effects of particulate matter air pollution in genetic type II diabetes mellitus. Part. Fibre Toxicol..

[23] Dubowsky S.D., Suh H., Schwartz J., Coull B.A., Gold D.R. (2006). Diabetes, Obesity, and Hypertension May Enhance Associations between Air Pollution and Markers of Systemic Inflammation. Environ. Health Perspect..

[24] Weinmayr G., Hennig F., Fuks K., Nonnemacher M., Jakobs H., Mohlenkamp S., Erbel R., Jockel K.H., Hoffmann B., Moebus S. (2015). Long-term exposure to fine particulate matter and incidence of type 2 diabetes mellitus in a cohort study: Effects of total and traffic-specific air pollution. Environ. Health.

[25] Rajagopalan S., Brook R.D. (2012). Air pollution and type 2 diabetes: Mechanistic insights. Diabetes.

[26] Jung C.R., Lin Y.T., Hwang B.F. (2015). Ozone, particulate matter, and newly diagnosed Alzheimer’s disease: A population-based cohort study in Taiwan. J. Alzheimer’s Dis..

[27] Moulton P.V., Yang W. (2012). Air pollution, oxidative stress, and Alzheimer’s disease. J. Environ. Public Health.

[28] Calderon-Garciduenas L., Franco-Lira M., Mora-Tiscareno A., Medina-Cortina H., Torres-Jardon R., Kavanaugh M. (2013). Early Alzheimer’s and Parkinson’s disease pathology in urban children: Friend versus Foe responses—It is time to face the evidence. BioMed Res. Int..

[29] Kirrane E.F., Bowman C., Davis J.A., Hoppin J.A., Blair A., Chen H., Patel M.M., Sandler D.P., Tanner C.M., Vinikoor-Imler L. (2015). Associations of Ozone and PM_2.5_ Concentrations With Parkinson’s Disease Among Participants in the Agricultural Health Study. J. Occup. Environ. Med..

[30] Heusinkveld H.J., Wahle T., Campbell A., Westerink R.H.S., Tran L., Johnston H., Stone V., Cassee F.R., Schins R.P.F. (2016). Neurodegenerative and neurological disorders by small inhaled particles. Neurotoxicology.

[31] Oberdorster G., Sharp Z., Atudorei V., Elder A., Gelein R., Kreyling W., Cox C. (2004). Translocation of inhaled ultrafine particles to the brain. Inhal. Toxicol..

[32] Park S.K., Adar S.D., O’Neill M.S., Auchincloss A.H., Szpiro A., Bertoni A.G., Navas-Acien A., Kaufman J.D., Diez-Roux A.V. (2015). Long-Term Exposure to Air Pollution and Type 2 Diabetes Mellitus in a Multiethnic Cohort. Am. J. Epidemiol..

[33] Guyatt G.H., Oxman A.D., Vist G.E., Kunz R., Falck-Ytter Y., Alonso-Coello P., Schunemann H.J., Group G.W. (2008). GRADE: An emerging consensus on rating quality of evidence and strength of recommendations. BMJ.

[34] Theorell T., Hammarstrom A., Aronsson G., Traskman Bendz L., Grape T., Hogstedt C., Marteinsdottir I., Skoog I., Hall C. (2015). A systematic review including meta-analysis of work environment and depressive symptoms. BMC Public Health.

[35] Morgan R.L., Thayer K.A., Bero L., Bruce N., Falck-Ytter Y., Ghersi D., Guyatt G., Hooijmans C., Langendam M., Mandrioli D. (2016). GRADE: Assessing the quality of evidence in environmental and occupational health. Environ. Int..

[36] Rooney A.A., Cooper G.S., Jahnke G.D., Lam J., Morgan R.L., Boyles A.L., Ratcliffe J.M., Kraft A.D., Schunemann H.J., Schwingl P. (2016). How credible are the study results? Evaluating and applying internal validity tools to literature-based assessments of environmental health hazards. Environ. Int..

[37] Tonne C., Elbaz A., Beevers S., Singh-Manoux A. (2014). Traffic-related air pollution in relation to cognitive function in older adults. Epidemiology.

[38] Chen J.C., Wang X., Wellenius G.A., Serre M.L., Driscoll I., Casanova R., McArdle J.J., Manson J.E., Chui H.C., Espeland M.A. (2015). Ambient air pollution and neurotoxicity on brain structure: Evidence from women’s health initiative memory study. Ann. Neurol..

[39] Power M.C., Weisskopf M.G., Alexeeff S.E., Coull B.A., Spiro A., Schwartz J. (2011). Traffic-related air pollution and cognitive function in a cohort of older men. Environ. Health Perspect..

[40] Ranft U., Schikowski T., Sugiri D., Krutmann J., Kramer U. (2009). Long-term exposure to traffic-related particulate matter impairs cognitive function in the elderly. Environ. Res..

[41] Schikowski T., Vossoughi M., Vierkotter A., Schulte T., Teichert T., Sugiri D., Fehsel K., Tzivian L., Bae I.S., Ranft U. (2015). Association of air pollution with cognitive functions and its modification by APOE gene variants in elderly women. Environ. Res..

[42] Weuve J., Puett R.C., Schwartz J., Yanosky J.D., Laden F., Grodstein F. (2012). Exposure to particulate air pollution and cognitive decline in older women. Arch. Intern. Med..

[43] Chen J.C., Schwartz J. (2009). Neurobehavioral effects of ambient air pollution on cognitive performance in US adults. Neurotoxicology.

[44] Kioumourtzoglou M.A., Schwartz J.D., Weisskopf M.G., Melly S.J., Wang Y., Dominici F., Zanobetti A. (2016). Long-term PM_2.5_ Exposure and Neurological Hospital Admissions in the Northeastern United States. Environ. Health Perspect..

[45] Tzivian L., Dlugaj M., Winkler A., Weinmayr G., Hennig F., Fuks K.B., Vossoughi M., Schikowski T., Weimar C., Erbel R. (2016). Long-term air pollution and traffic noise exposures and mild cognitive impairment in older adults: A cross-sectional analysis of the Heinz Nixdorf recall study. Environ. Health Perspect..

[46] Chen C.Y., Hung H.J., Chang K.H., Hsu C.Y., Muo C.H., Tsai C.H., Wu T.N. (2017). Long-term exposure to air pollution and the incidence of Parkinson’s disease: A nested case-control study. PLoS ONE.

[47] Ailshire J.A., Clarke P. (2015). Fine particulate matter air pollution and cognitive function among U.S. older adults. J. Gerontol. B Psychol. Sci. Soc. Sci..

[48] Wu Y.C., Lin Y.C., Yu H.L., Chen J.H., Chen T.F., Sun Y., Wen L.L., Yip P.K., Chu Y.M., Chen Y.C. (2015). Association between air pollutants and dementia risk in the elderly. Alzheimer’s Dement..

[49] Palacios N., Fitzgerald K.C., Hart J.E., Weisskopf M., Schwarzschild M.A., Ascherio A., Laden F. (2017). Air Pollution and Risk of Parkinson’s Disease in a Large Prospective Study of Men. Environ. Health Perspect..

[50] Palacios N., Fitzgerald K.C., Hart J.E., Weisskopf M.G., Schwarzschild M.A., Ascherio A., Laden F. (2014). Particulate matter and risk of Parkinson disease in a large prospective study of women. Environ. Health.

[51] Liu R., Young M.T., Chen J.C., Kaufman J.D., Chen H. (2016). Ambient Air Pollution Exposures and Risk of Parkinson Disease. Environ. Health Perspect..

[52] Power M.C., Adar S.D., Yanosky J.D., Weuve J. (2016). Exposure to air pollution as a potential contributor to cognitive function, cognitive decline, brain imaging, and dementia: A systematic review of epidemiologic research. Neurotoxicology.

[53] Ferreira L.K., Busatto G.F. (2011). Neuroimaging in Alzheimer’s disease: Current role in clinical practice and potential future applications. Clinics.

[54] Chen H., Kwong J.C., Copes R., Tu K., Villeneuve P.J., van Donkelaar A., Hystad P., Martin R.V., Murray B.J., Jessiman B. (2017). Living near major roads and the incidence of dementia, Parkinson’s disease, and multiple sclerosis: A population-based cohort study. Lancet.

[55] Sharp E.S., Gatz M. (2011). Relationship between education and dementia: An updated systematic review. Alzheimer Dis. Assoc. Disord..

[56] Fischer C., Yeung E., Hansen T., Gibbons S., Fornazzari L., Ringer L., Schweizer T.A. (2009). Impact of socioeconomic status on the prevalence of dementia in an inner city memory disorders clinic. Int. Psychogeriatr..

[57] Russ T.C., Stamatakis E., Hamer M., Starr J.M., Kivimaki M., Batty G.D. (2013). Socioeconomic status as a risk factor for dementia death: Individual participant meta-analysis of 86,508 men and women from the UK. Br. J. Psychiatry.

[58] Liu C., Yang C., Zhao Y., Ma Z., Bi J., Liu Y., Meng X., Wang Y., Cai J., Chen R. (2016). Associations between long-term exposure to ambient particulate air pollution and type 2 diabetes prevalence, blood glucose and glycosylated hemoglobin levels in China. Environ. Int..

[59] Wolf K., Popp A., Schneider A., Breitner S., Hampel R., Rathmann W., Herder C., Roden M., Koenig W., Meisinger C. (2016). Association Between Long-term Exposure to Air Pollution and Biomarkers Related to Insulin Resistance, Subclinical Inflammation, and Adipokines. Diabetes.

[60] Eze I.C., Schaffner E., Foraster M., Imboden M., von Eckardstein A., Gerbase M.W., Rothe T., Rochat T., Kunzli N., Schindler C. (2015). Long-Term Exposure to Ambient Air Pollution and Metabolic Syndrome in Adults. PLoS ONE.

[61] O’Donovan G., Chudasama Y., Grocock S., Leigh R., Dalton A.M., Gray L.J., Yates T., Edwardson C., Hill S., Henson J. (2017). The association between air pollution and type 2 diabetes in a large cross-sectional study in Leicester: The CHAMPIONS Study. Environ. Int..

[62] De Sio S., Rosati M.V., Cherubini E., Ciarrocca M., Baccolo T.P., Grimaldi F., Caciari T., Tomao E., Tomei F. (2005). Occupational exposure to urban pollutants and plasma insulin. Saudi Med. J..

[63] Brook R.D., Cakmak S., Turner M.C., Brook J.R., Crouse D.L., Peters P.A., van Donkelaar A., Villeneuve P.J., Brion O., Jerrett M. (2013). Long-term fine particulate matter exposure and mortality from diabetes in Canada. Diabetes Care.

[64] Li C., Fang D., Xu D., Wang B., Zhao S., Yan S., Wang Y. (2014). Main air pollutants and diabetes-associated mortality: A systematic review and meta-analysis. Eur. J. Endocrinol..

[65] Pope C.A., Turner M.C., Burnett R.T., Jerrett M., Gapstur S.M., Diver W.R., Krewski D., Brook R.D. (2015). Relationships between fine particulate air pollution, cardiometabolic disorders, and cardiovascular mortality. Circ. Res..

[66] Chen H., Burnett R.T., Kwong J.C., Villeneuve P.J., Goldberg M.S., Brook R.D., van Donkelaar A., Jerrett M., Martin R.V., Brook J.R. (2013). Risk of incident diabetes in relation to long-term exposure to fine particulate matter in Ontario, Canada. Environ. Health Perspect..

[67] Hansen A.B., Ravnskjaer L., Loft S., Andersen K.K., Brauner E.V., Baastrup R., Yao C., Ketzel M., Becker T., Brandt J. (2016). Long-term exposure to fine particulate matter and incidence of diabetes in the Danish Nurse Cohort. Environ. Int..

[68] Lyons T.J., Basu A. (2012). Biomarkers in diabetes: Hemoglobin A1c, vascular and tissue markers. Transl. Res..

[69] Chen Z., Salam M.T., Toledo-Corral C., Watanabe R.M., Xiang A.H., Buchanan T.A., Habre R., Bastain T.M., Lurmann F., Wilson J.P. (2016). Ambient Air Pollutants Have Adverse Effects on Insulin and Glucose Homeostasis in Mexican Americans. Diabetes Care.

[70] Kramer U., Herder C., Sugiri D., Strassburger K., Schikowski T., Ranft U., Rathmann W. (2010). Traffic-related air pollution and incident type 2 diabetes: Results from the SALIA cohort study. Environ. Health Perspect..

[71] Kim J.H., Hong Y.C. (2012). GSTM1, GSTT1, and GSTP1 polymorphisms and associations between air pollutants and markers of insulin resistance in elderly Koreans. Environ. Health Perspect..

[72] Puett R.C., Hart J.E., Schwartz J., Hu F.B., Liese A.D., Laden F. (2011). Are particulate matter exposures associated with risk of type 2 diabetes?. Environ. Health Perspect..

[73] Chen L., Zhou Y., Li S., Williams G., Kan H., Marks G.B., Morawska L., Abramson M.J., Chen S., Yao T. (2016). Air pollution and fasting blood glucose: A longitudinal study in China. Sci. Total Environ..

[74] Wang Y., Eliot M.N., Kuchel G.A., Schwartz J., Coull B.A., Mittleman M.A., Lipsitz L.A., Wellenius G.A. (2014). Long-term exposure to ambient air pollution and serum leptin in older adults: Results from the MOBILIZE Boston study. J. Occup. Environ. Med..

[75] Eze I.C., Hemkens L.G., Bucher H.C., Hoffmann B., Schindler C., Kunzli N., Schikowski T., Probst-Hensch N.M. (2015). Association between ambient air pollution and diabetes mellitus in Europe and North America: Systematic review and meta-analysis. Environ. Health Perspect..

[76] Guyatt G.H., Oxman A.D., Montori V., Vist G., Kunz R., Brozek J., Alonso-Coello P., Djulbegovic B., Atkins D., Falck-Ytter Y. (2011). GRADE guidelines: 5. Rating the quality of evidence—Publication bias. J. Clin. Epidemiol..

[77] Guyatt G.H., Oxman A.D., Kunz R., Woodcock J., Brozek J., Helfand M., Alonso-Coello P., Falck-Ytter Y., Jaeschke R., Vist G. (2011). GRADE guidelines: 8. Rating the quality of evidence—Indirectness. J. Clin. Epidemiol..

[78] Clifford A., Lang L., Chen R., Anstey K.J., Seaton A. (2016). Exposure to air pollution and cognitive functioning across the life course—A systematic literature review. Environ. Res..

[79] Palacios N., Fitzgerald K., Roberts A.L., Hart J.E., Weisskopf M.G., Schwarzschild M.A., Ascherio A., Laden F. (2014). A prospective analysis of airborne metal exposures and risk of Parkinson disease in the nurses’ health study cohort. Environ. Health Perspect..

[80] Ailshire J.A., Crimmins E.M. (2014). Fine particulate matter air pollution and cognitive function among older US adults. Am. J. Epidemiol..

[81] Fung K.Y., Luginaah I.N., Gorey K.M. (2007). Impact of air pollution on hospital admissions in Southwestern Ontario, Canada: Generating hypotheses in sentinel high-exposure places. Environ Health.

[82] Kunzli N., Tager I.B. (2005). Air pollution: From lung to heart. Swiss Med. Wkly..

[83] Janghorbani M., Momeni F., Mansourian M. (2014). Systematic review and meta-analysis of air pollution exposure and risk of diabetes. Eur. J. Epidemiol..

[84] Thibault V., Belanger M., LeBlanc E., Babin L., Halpine S., Greene B., Mancuso M. (2016). Factors that could explain the increasing prevalence of type 2 diabetes among adults in a Canadian province: A critical review and analysis. Diabetol. Metab. Syndr..

[85] Robertson M., Seaton A., Whalley L.J. (2015). Can we reduce the risk of dementia?. QJM.

[86] Baldi I., Lebailly P., Mohammed-Brahim B., Letenneur L., Dartigues J.F., Brochard P. (2003). Neurodegenerative diseases and exposure to pesticides in the elderly. Am. J. Epidemiol..

[87] Donaldson K., Brown D., Clouter A., Duffin R., MacNee W., Renwick L., Tran L., Stone V. (2002). The pulmonary toxicology of ultrafine particles. J. Aerosol Med..

[88] Johnston H.J., Verdon R., Gillies S., Brown D.M., Fernandes T.F., Henry T.B., Rossi A.G., Tran L., Tucker C., Tyler C.R. (2018). Adoption of in vitro systems and zebrafish embryos as alternative models for reducing rodent use in assessments of immunological and oxidative stress responses to nanomaterials. Crit. Rev. Toxicol..

[89] Duncan B.B., Schmidt M.I., Pankow J.S., Ballantyne C.M., Couper D., Vigo A., Hoogeveen R., Folsom A.R., Heiss G., Atherosclerosis Risk in Communities (2003). Low-grade systemic inflammation and the development of type 2 diabetes: The atherosclerosis risk in communities study. Diabetes.

[90] Wellen K.E., Hotamisligil G.S. (2005). Inflammation, stress, and diabetes. J. Clin. Investig..

[91] Rehman K., Akash M.S. (2016). Mechanisms of inflammatory responses and development of insulin resistance: How are they interlinked?. J. Biomed. Sci..

[92] Meigs J.B. (2009). Multiple biomarker prediction of type 2 diabetes. Diabetes Care.

[93] Rioux C.L., Tucker K.L., Brugge D., Gute D.M., Mwamburi M. (2011). Traffic exposure in a population with high prevalence type 2 diabetes—Do medications influence concentrations of C-reactive protein?. Environ. Pollut..

[94] Khafaie M.A., Salvi S.S., Ojha A., Khafaie B., Gore S.S., Yajnik C.S. (2013). Systemic inflammation (C-reactive protein) in type 2 diabetic patients is associated with ambient air pollution in Pune City, India. Diabetes Care.

[95] Yang B.Y., Qian Z.M., Li S., Chen G., Bloom M.S., Elliott M., Syberg K.W., Heinrich J., Markevych I., Wang S.Q. (2018). Ambient air pollution in relation to diabetes and glucose-homoeostasis markers in China: A cross-sectional study with findings from the 33 Communities Chinese Health Study. Lancet Planet Health.

[96] Bolos M., Perea J.R., Avila J. (2017). Alzheimer’s disease as an inflammatory disease. Biomol. Concepts.

[97] De Groot N.S., Burgas M.T. (2015). Is membrane homeostasis the missing link between inflammation and neurodegenerative diseases?. Cell Mol. Life Sci..

[98] Morales I., Farias G., Maccioni R.B. (2010). Neuroimmunomodulation in the pathogenesis of Alzheimer’s disease. Neuroimmunomodulation.

[99] Shepherd C., McCann H., Halliday G.M. (2009). Variations in the neuropathology of familial Alzheimer’s disease. Acta Neuropathol..

[100] Gupta A., Watkins A., Thomas P., Majer R., Habubi N., Morris G., Pansari K. (2005). Coagulation and inflammatory markers in Alzheimer’s and vascular dementia. Int. J. Clin. Pract..

[101] Koyama A., O’Brien J., Weuve J., Blacker D., Metti A.L., Yaffe K. (2013). The role of peripheral inflammatory markers in dementia and Alzheimer’s disease: A meta-analysis. J. Gerontol. Ser. A Biol. Sci. Med. Sci..

[102] Jefferson A.L., Massaro J.M., Wolf P.A., Seshadri S., Au R., Vasan R.S., Larson M.G., Meigs J.B., Keaney J.F., Lipinska I. (2007). Inflammatory biomarkers are associated with total brain volume: The Framingham Heart Study. Neurology.

[103] Calderon-Garciduenas L., Reed W., Maronpot R.R., Henriquez-Roldan C., Delgado-Chavez R., Calderon-Garciduenas A., Dragustinovis I., Franco-Lira M., Aragon-Flores M., Solt A.C. (2004). Brain inflammation and Alzheimer’s-like pathology in individuals exposed to severe air pollution. Toxicol. Pathol..

[104] Calderon-Garciduenas L., Azzarelli B., Acuna H., Garcia R., Gambling T.M., Osnaya N., Monroy S., Del Tizapantzi M.R., Carson J.L., Villarreal-Calderon A. (2002). Air pollution and brain damage. Toxicol. Pathol..

[105] Calderon-Garciduenas L., Maronpot R.R., Torres-Jardon R., Henriquez-Roldan C., Schoonhoven R., Acuna-Ayala H., Villarreal-Calderon A., Nakamura J., Fernando R., Reed W. (2003). DNA damage in nasal and brain tissues of canines exposed to air pollutants is associated with evidence of chronic brain inflammation and neurodegeneration. Toxicol. Pathol..

[106] Campbell A., Oldham M., Becaria A., Bondy S., Meacher D., Sioutas C., Misra C., Mendez L., Kleinman M. (2005). Particulate matter in polluted air may increase biomarkers of inflammation in mouse brain. Neurotoxicology.

[107] Gerlofs-Nijland M.E., van Berlo D., Cassee F.R., Schins R.P., Wang K., Campbell A. (2010). Effect of prolonged exposure to diesel engine exhaust on proinflammatory markers in different regions of the rat brain. Part. Fibre Toxicol..

[108] Durga M., Devasena T., Rajasekar A. (2015). Determination of LC50 and sub-chronic neurotoxicity of diesel exhaust nanoparticles. Environ. Toxicol. Pharmacol..

[109] Cole T.B., Coburn J., Dao K., Roque P., Chang Y.C., Kalia V., Guilarte T.R., Dziedzic J., Costa L.G. (2016). Sex and genetic differences in the effects of acute diesel exhaust exposure on inflammation and oxidative stress in mouse brain. Toxicology.

[110] Kim S.Y., Kim J.K., Park S.H., Kim B.G., Jang A.S., Oh S.H., Lee J.H., Suh M.W., Park M.K. (2018). Effects of inhaled particulate matter on the central nervous system in mice. Neurotoxicology.

[111] Hullmann M., Albrecht C., van Berlo D., Gerlofs-Nijland M.E., Wahle T., Boots A.W., Krutmann J., Cassee F.R., Bayer T.A., Schins R.P.F. (2017). Diesel engine exhaust accelerates plaque formation in a mouse model of Alzheimer’s disease. Part. Fibre Toxicol..

[112] Morgan T.E., Davis D.A., Iwata N., Tanner J.A., Snyder D., Ning Z., Kam W., Hsu Y.T., Winkler J.W., Chen J.C. (2011). Glutamatergic neurons in rodent models respond to nanoscale particulate urban air pollutants in vivo and in vitro. Environ. Health Perspect..

[113] Solaimani P., Saffari A., Sioutas C., Bondy S.C., Campbell A. (2017). Exposure to ambient ultrafine particulate matter alters the expression of genes in primary human neurons. Neurotoxicology.

[114] Gillespie P., Tajuba J., Lippmann M., Chen L.C., Veronesi B. (2013). Particulate matter neurotoxicity in culture is size-dependent. Neurotoxicology.

[115] Campbell A., Daher N., Solaimani P., Mendoza K., Sioutas C. (2014). Human brain derived cells respond in a type-specific manner after exposure to urban particulate matter (PM). Toxicol. In Vitro.

[116] Goldman S.M. (2014). Environmental toxins and Parkinson’s disease. Annu. Rev. Pharmacol. Toxicol..

[117] Niranjan R. (2014). The role of inflammatory and oxidative stress mechanisms in the pathogenesis of Parkinson’s disease: Focus on astrocytes. Mol. Neurobiol..

[118] Allen K.V., Frier B.M., Strachan M.W. (2004). The relationship between type 2 diabetes and cognitive dysfunction: Longitudinal studies and their methodological limitations. Eur. J. Pharmacol..

[119] Barbieri M., Boccardi V., Paolisso G., Martin C.R., Preedy V.R. (2015). Chapter 35—Cognitive Decline and Diabetes: A Focus on Linking Mechanisms. Diet and Nutrition in Dementia and Cognitive Decline.

[120] Craft S. (2005). Insulin resistance syndrome and Alzheimer’s disease: Age- and obesity-related effects on memory, amyloid, and inflammation. Neurobiol. Aging.

[121] Accardi G., Caruso C., Colonna-Romano G., Camarda C., Monastero R., Candore G. (2012). Can Alzheimer disease be a form of type 3 diabetes?. Rejuvenat. Res..

[122] Cheng G., Huang C., Deng H., Wang H. (2012). Diabetes as a risk factor for dementia and mild cognitive impairment: A meta-analysis of longitudinal studies. Intern. Med. J..

[123] Frisardi V., Solfrizzi V., Seripa D., Capurso C., Santamato A., Sancarlo D., Vendemiale G., Pilotto A., Panza F. (2010). Metabolic-cognitive syndrome: A cross-talk between metabolic syndrome and Alzheimer’s disease. Ageing Res. Rev..

[124] Garcia-Lara J.M., Aguilar-Navarro S., Gutierrez-Robledo L.M., Avila-Funes J.A. (2010). The metabolic syndrome, diabetes, and Alzheimer’s disease. Revista de Investigacion Clinica Organo del Hospital de Enfermedades de la Nutricion.

[125] Ohara T., Doi Y., Ninomiya T., Hirakawa Y., Hata J., Iwaki T., Kanba S., Kiyohara Y. (2011). Glucose tolerance status and risk of dementia in the community: The Hisayama study. Neurology.

[126] Adeghate E., Donath T., Adem A. (2013). Alzheimer disease and diabetes mellitus: Do they have anything in common?. Curr. Alzheimer Res..

[127] Alam F., Islam M.A., Sasongko T.H., Gan S.H. (2016). Type 2 Diabetes Mellitus and Alzheimer’s Disease: Bridging the Pathophysiology and Management. Curr. Pharm. Des..

[128] D’Amelio M., Ragonese P., Callari G., Di Benedetto N., Palmeri B., Terruso V., Salemi G., Famoso G., Aridon P., Savettieri G. (2009). Diabetes preceding Parkinson’s disease onset. A case-control study. Parkinsonism Relat. Disord..

[129] Hu G., Jousilahti P., Bidel S., Antikainen R., Tuomilehto J. (2007). Type 2 diabetes and the risk of Parkinson’s disease. Diabetes Care.

[130] Yue X., Li H., Yan H., Zhang P., Chang L., Li T. (2016). Risk of Parkinson Disease in Diabetes Mellitus: An Updated Meta-Analysis of Population-Based Cohort Studies. Medicine.

[131] van Himbergen T.M., Beiser A.S., Ai M., Seshadri S., Otokozawa S., Au R., Thongtang N., Wolf P.A., Schaefer E.J. (2012). Biomarkers for insulin resistance and inflammation and the risk for all-cause dementia and alzheimer disease: Results from the Framingham Heart Study. Arch. Neurol..

[132] Jayaraman A., Pike C.J. (2014). Alzheimer’s disease and type 2 diabetes: Multiple mechanisms contribute to interactions. Curr. Diabetes Rep..

[133] Cardoso S., Correia S., Santos R.X., Carvalho C., Santos M.S., Oliveira C.R., Perry G., Smith M.A., Zhu X., Moreira P.I. (2009). Insulin is a two-edged knife on the brain. J. Alzheimer’s Dis..

[134] Kleinridders A., Ferris H.A., Cai W., Kahn C.R. (2014). Insulin action in brain regulates systemic metabolism and brain function. Diabetes.

[135] Verdile G., Keane K.N., Cruzat V.F., Medic S., Sabale M., Rowles J., Wijesekara N., Martins R.N., Fraser P.E., Newsholme P. (2015). Inflammation and Oxidative Stress: The Molecular Connectivity between Insulin Resistance, Obesity, and Alzheimer’s Disease. Mediat. Inflamm..

[136] Diaz J., Martinez-Martin P., Rodriguez-Blazquez C., Vazquez B., Forjaz M.J., Ortiz C., Carmona R., Linares C. (2017). Short-term association between road traffic noise and healthcare demand generated by Parkinson’s disease in Madrid, Spain. Gac. Sanit..

[137] Cui B., Li K., Gai Z., She X., Zhang N., Xu C., Chen X., An G., Ma Q., Wang R. (2015). Chronic Noise Exposure Acts Cumulatively to Exacerbate Alzheimer’s Disease-Like Amyloid-beta Pathology and Neuroinflammation in the Rat Hippocampus. Sci. Rep..

[138] Bakre A.T., Chen R., Khutan R., Wei L., Smith T., Qin G., Danat I.M., Zhou W., Schofield P., Clifford A. (2018). Association between fish consumption and risk of dementia: A new study from China and a systematic literature review and meta-analysis. Public Health Nutr..

[139] Balti E.V., Echouffo-Tcheugui J.B., Yako Y.Y., Kengne A.P. (2014). Air pollution and risk of type 2 diabetes mellitus: A systematic review and meta-analysis. Diabetes Res. Clin. Pract..

[140] Oberdurster G. (2000). Toxicology of ultrafine particles: In vivo studies. Philos. Trans. R. Soc. A.

[141] Zhang R., Dai Y., Zhang X., Niu Y., Meng T., Li Y., Duan H., Bin P., Ye M., Jia X. (2014). Reduced pulmonary function and increased pro-inflammatory cytokines in nanoscale carbon black-exposed workers. Part. Fibre Toxicol..

[142] Castranova V., Frazer D.G., Manley L.K., Dey R.D. (2002). Pulmonary alterations associated with inhalation of occupational and environmental irritants. Int. Immunopharmacol..

